# 3D-Beacons: decreasing the gap between protein sequences and structures through a federated network of protein structure data resources

**DOI:** 10.1093/gigascience/giac118

**Published:** 2022-11-30

**Authors:** Mihaly Varadi, Sreenath Nair, Ian Sillitoe, Gerardo Tauriello, Stephen Anyango, Stefan Bienert, Clemente Borges, Mandar Deshpande, Tim Green, Demis Hassabis, Andras Hatos, Tamas Hegedus, Maarten L Hekkelman, Robbie Joosten, John Jumper, Agata Laydon, Dmitry Molodenskiy, Damiano Piovesan, Edoardo Salladini, Steven L Salzberg, Markus J Sommer, Martin Steinegger, Erzsebet Suhajda, Dmitri Svergun, Luiggi Tenorio-Ku, Silvio Tosatto, Kathryn Tunyasuvunakool, Andrew Mark Waterhouse, Augustin Žídek, Torsten Schwede, Christine Orengo, Sameer Velankar

**Affiliations:** European Molecular Biology Laboratory, European Bioinformatics Institute, Hinxton CB10 1SA, UK; European Molecular Biology Laboratory, European Bioinformatics Institute, Hinxton CB10 1SA, UK; Department of Structural and Molecular Biology, UCL, London WC1E 6BT, UK; Biozentrum, University of Basel, Basel 4056, Switzerland; Computational Structural Biology, SIB Swiss Institute of Bioinformatics, Basel 4056, Switzerland; European Molecular Biology Laboratory, European Bioinformatics Institute, Hinxton CB10 1SA, UK; Biozentrum, University of Basel, Basel 4056, Switzerland; Computational Structural Biology, SIB Swiss Institute of Bioinformatics, Basel 4056, Switzerland; Computational Structural Biology, SIB Swiss Institute of Bioinformatics, Basel 4056, Switzerland; European Molecular Biology Laboratory, EMBL Hamburg, Hamburg 69117, Germany; European Molecular Biology Laboratory, European Bioinformatics Institute, Hinxton CB10 1SA, UK; DeepMind, London EC4A 3TW, UK; DeepMind, London EC4A 3TW, UK; Department of Biomedical Sciences, University of Padova, Padova 35129, Italy; Department of Oncology, Lausanne University Hospital, Lausanne 1015, Switzerland; Department of Computational Biology, University of Lausanne, Lausanne 1015, Switzerland; Swiss Institute of Bioinformatics, Lausanne 1015, Switzerland; Swiss Cancer Center Leman, Lausanne 1005, Switzerland; Department of Biophysics and Radiation Biology, Semmelweis University, Budapest 1094, Hungary; Netherlands Cancer Institute, Amsterdam 1066 CX, The Netherlands; Netherlands Cancer Institute, Amsterdam 1066 CX, The Netherlands; DeepMind, London EC4A 3TW, UK; DeepMind, London EC4A 3TW, UK; Computational Structural Biology, SIB Swiss Institute of Bioinformatics, Basel 4056, Switzerland; European Molecular Biology Laboratory, EMBL Hamburg, Hamburg 69117, Germany; Department of Biomedical Sciences, University of Padova, Padova 35129, Italy; Department of Biomedical Sciences, University of Padova, Padova 35129, Italy; Biomedical Engineering, Johns Hopkins University, Baltimore, MD 21205, USA; Biomedical Engineering, Johns Hopkins University, Baltimore, MD 21205, USA; School of Biology, Seoul National University, Seoul 82-2-880-6971, 6977, South Korea; Department of Biophysics and Radiation Biology, Semmelweis University, Budapest 1094, Hungary; Computational Structural Biology, SIB Swiss Institute of Bioinformatics, Basel 4056, Switzerland; European Molecular Biology Laboratory, EMBL Hamburg, Hamburg 69117, Germany; Department of Biomedical Sciences, University of Padova, Padova 35129, Italy; Department of Biomedical Sciences, University of Padova, Padova 35129, Italy; DeepMind, London EC4A 3TW, UK; Biozentrum, University of Basel, Basel 4056, Switzerland; Computational Structural Biology, SIB Swiss Institute of Bioinformatics, Basel 4056, Switzerland; DeepMind, London EC4A 3TW, UK; Biozentrum, University of Basel, Basel 4056, Switzerland; Computational Structural Biology, SIB Swiss Institute of Bioinformatics, Basel 4056, Switzerland; Department of Structural and Molecular Biology, UCL, London WC1E 6BT, UK; European Molecular Biology Laboratory, European Bioinformatics Institute, Hinxton CB10 1SA, UK

**Keywords:** structural biology, experimentally determined structures computationally predicted structures, federated data network, bioinformatics

## Abstract

While scientists can often infer the biological function of proteins from their 3-dimensional quaternary structures, the gap between the number of known protein sequences and their experimentally determined structures keeps increasing. A potential solution to this problem is presented by ever more sophisticated computational protein modeling approaches. While often powerful on their own, most methods have strengths and weaknesses. Therefore, it benefits researchers to examine models from various model providers and perform comparative analysis to identify what models can best address their specific use cases. To make data from a large array of model providers more easily accessible to the broader scientific community, we established 3D-Beacons, a collaborative initiative to create a federated network with unified data access mechanisms. The 3D-Beacons Network allows researchers to collate coordinate files and metadata for experimentally determined and theoretical protein models from state-of-the-art and specialist model providers and also from the Protein Data Bank.

## Introduction

Proteins are essential building blocks of almost every biological process; therefore, understanding their functions is critical to many applications, from drug discovery [1, 2] to tackling environmental challenges such as plastic pollution [3]. Accurate information on the structure of a protein, especially in the context of its biological assembly, can help scientists understand and modulate its function [4, 5].

**Figure 1:**
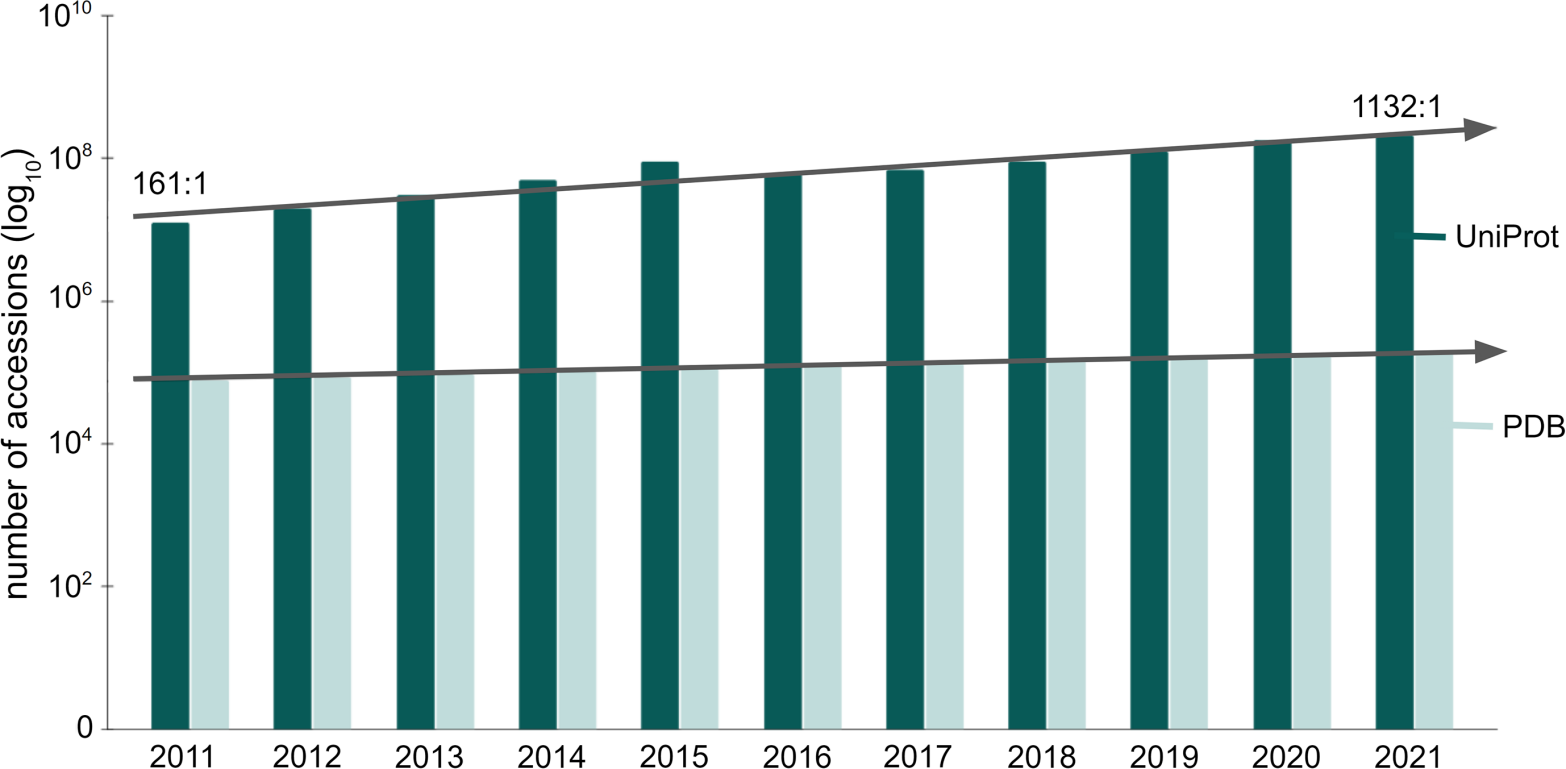
Growth of the UniProt and the PDB databases. This figure shows the number of accessions (on a logarithmic scale) throughout the past decade. In 2011, the UniProt had 161× as many protein sequences as the number of PDB entries. This ratio grew by an order of magnitude and was 1,132 to 1 in 2021, showing that the gap between known protein sequences and their structures keeps increasing.

Unfortunately, gaining such insights regarding the function of proteins through their structures is severely hampered by the lack of high-quality, experimentally determined structures. As of 2022, the Universal Protein Resource (UniProt) contains around 204 million nonredundant amino acid sequences, while the Protein Data Bank (PDB) [6, 7] contains around 190,000 PDB entries mapped to approximately 52,000 UniProt accessions. In other words, less than 0.03% of all the known protein sequences have experimentally determined atomic resolution structures. As sequencing becomes more accessible, the gap between protein sequences and structures increases (Fig. 1).

A practical approach to addressing this challenge relies on high-accuracy computational models to complement the experimentally determined structures when the latter are unavailable for a certain protein of interest [8]. The thermodynamic hypothesis postulates that within certain limitations, the native structure is determined only by the protein's amino acid sequence [9, 10]. Indeed, the past 50 years saw the development of many algorithms and scientific software to predict protein structures [11, 12]. An approach developed early in this field was to use homologous protein structures as templates. Several modeling tools and data resources have long provided access to such models, for example, the SWISS-MODEL and the ModBase web services and databases [13–15]. In 2021, the field saw tremendous advances with tools such as AlphaFold and RoseTTAFold achieving much higher accuracy for *de novo* predictions without homologous templates than ever before [16, 17]. This new generation of prediction tools makes it possible to try and predict the structure of virtually any known protein based on its sequence.

While these new techniques are increasingly accurate, it is important that they are supplemented with reliable estimates of model confidence both for the whole model and locally for each residue. Researchers should not expect all predictions to be equally accurate neither globally nor in every region, and confidence estimates should hence be used to determine if a predicted structure can be used for downstream analysis [18]. Commonly used model confidence methods aim to predict the global and local similarity of the model compared to the correct coordinates if those coordinates were provided by an experimentally determined structure. In recent years, several model prediction methods such as SWISS-MODEL [14], RoseTTAFold [17], and AlphaFold [16] have chosen the superposition-free local distance difference test (lDDT) score [19] as a similarity metric to provide model confidence for their own models. The lDDT score measures differences in interatomic distances within a short radius between model and reference structure. It has been shown that superposition-free measures are robust with respect to domain movements and have advantages for the analysis of local structural details [20]. Similarly, superposition-free measures have been used for a long time in the creation of experimental structure models [21].

**Figure 2:**
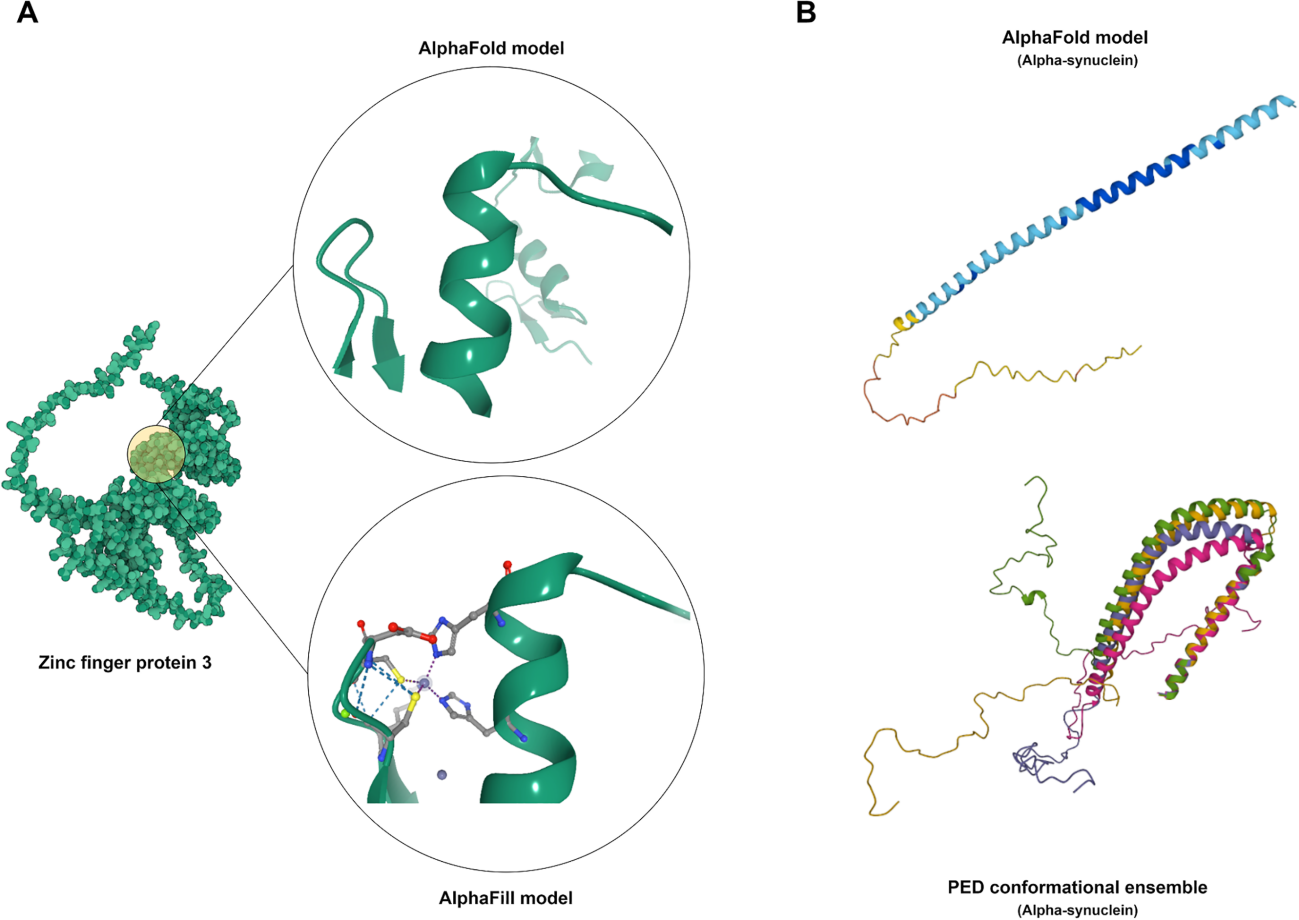
Highlighting the strengths and weaknesses of modeling techniques. Each modeling approach has limitations and specific strengths. For example, AlphaFill complements AlphaFold models by placing obligate ligands in their contexts (A). Other data providers, such as the Protein Ensemble Database, provide conformational ensembles for intrinsically disordered proteins (IDPs), for example, for the human Alpha-synuclein (B).

Another important consideration when relying on any structure prediction tool is to consider its limitations. While structures in the PDB have the advantage of experimental data backing the coordinates, enabling experimental as well as geometric validation, it is a relatively small dataset, as discussed above. Template-based models have the distinct advantage of enabling the mapping of a model to homologues with known structures, thus mapping to experimentally derived structures that can be in distinct conformational states or in complex with other molecules. Some tools excel at general-purpose protein structure modeling; others specialize in placing relevant ligands in the context of a model or representing conformational flexibility with ensembles of potential conformations [14, 16, 22–24] (Fig. 2). For example, AlphaFold 2.0 cannot perform docking of small molecules, even if they are obligate ligands of the proteins, such as Zinc-finger proteins. However, data resources such as AlphaFill can tackle this problem by building on existing models and adding known ligands to these structures [23] (Fig. 2A). On the other hand, the central repository of AlphaFold models, the AlphaFold Structure Database, only contains predictions for single polypeptide chains and not necessarily the functional forms of proteins [25]. In the case of multimeric complexes, the functional form can include several polypeptide chains. Since the number of known protein complexes is immense, having a comprehensive database for complex structures soon is rather unlikely. Therefore, integrating 3-dimensional data from experts in specialized fields of proteins is important, as demonstrated by physiologically and pathologically relevant transmembrane ABC half transporters [26] and by a set of computed structures of core eukaryotic protein complexes deposited in the ModelArchive [27]. Databases such as the Small-Angle Scattering Biological Data Bank (SASBDB) [28] and the Protein Ensemble Database (PED) [22] highlight the dynamic nature of intrinsically disordered proteins (Fig. 2B). Small-angle scattering provides low-resolution information on the shape and size of biological macromolecules in solution, but it also offers powerful means for the quantitative analysis of flexible systems, including intrinsically disordered proteins (IDPs) [29]. Theses data, together with *ab initio* modeling approaches, can be used to generate an experimentally validated pool of IDP models. PED provides access to such conformational ensembles but also those based on other experimental approaches. Considering the limitations of certain tools highlights the importance of using models and methods from various synergistic software and data providers to mitigate the weaknesses of individual modeling techniques.

While many prediction software and several publicly accessible data resources host and archive protein structures, these resources are fragmented and often rely on their own data standards to describe the necessary meta-information essential for providing context for a specific model. They also offer distinct data access mechanisms, requiring the users to learn multiple sets of technical details when interacting with various resources. The lack of standardization can severely impede the comparative analysis of these models, making it difficult to gain valuable insights.

Here, we present the 3D-Beacons Network, an open, collaborative platform for providing programmatic access to 3-dimensional coordinates and their standardized meta-information from both experimentally determined and computationally modeled protein structures.

## Results

The 3D-Beacons Network is an open collaboration between providers of experimentally determined and computationally predicted protein structures. To date, 10 data providers make their protein structures available through this platform (Table 1). The consortium is guided by a collaboration agreement that prospective data providers agree to comply with. We encourage and invite macromolecular structure providers from research teams focusing on small, curated datasets to large data resources to join the 3D-Beacons Network and take advantage of its infrastructure to make their models more accessible to the scientific community. Importantly, all the data provided through the network must be freely available for academic and commercial use under Creative Commons Attribution 4.0 license terms.

**Table 1:** Members of the 3D-Beacons Network

Data provider	Model category	Number of structures*
AlphaFill	Template based	995,411
AlphaFold DB	*Ab initio*	214,684,311
Genome3D	Template based	*In progress*
HegeLab	*Ab initio*	15
isoform.io	*Ab initio*	48,551
ModelArchive	*Ab initio*/template based	1,106
PDBe	Experimentally determined	190,639
PED	Conformation ensembles	275
SASBDB	Experimentally determined	3,912
SWISS-MODEL Repository	Template based	2,216,915

* Numbers are accurate as of 29 July 2022.

**Figure 3:**
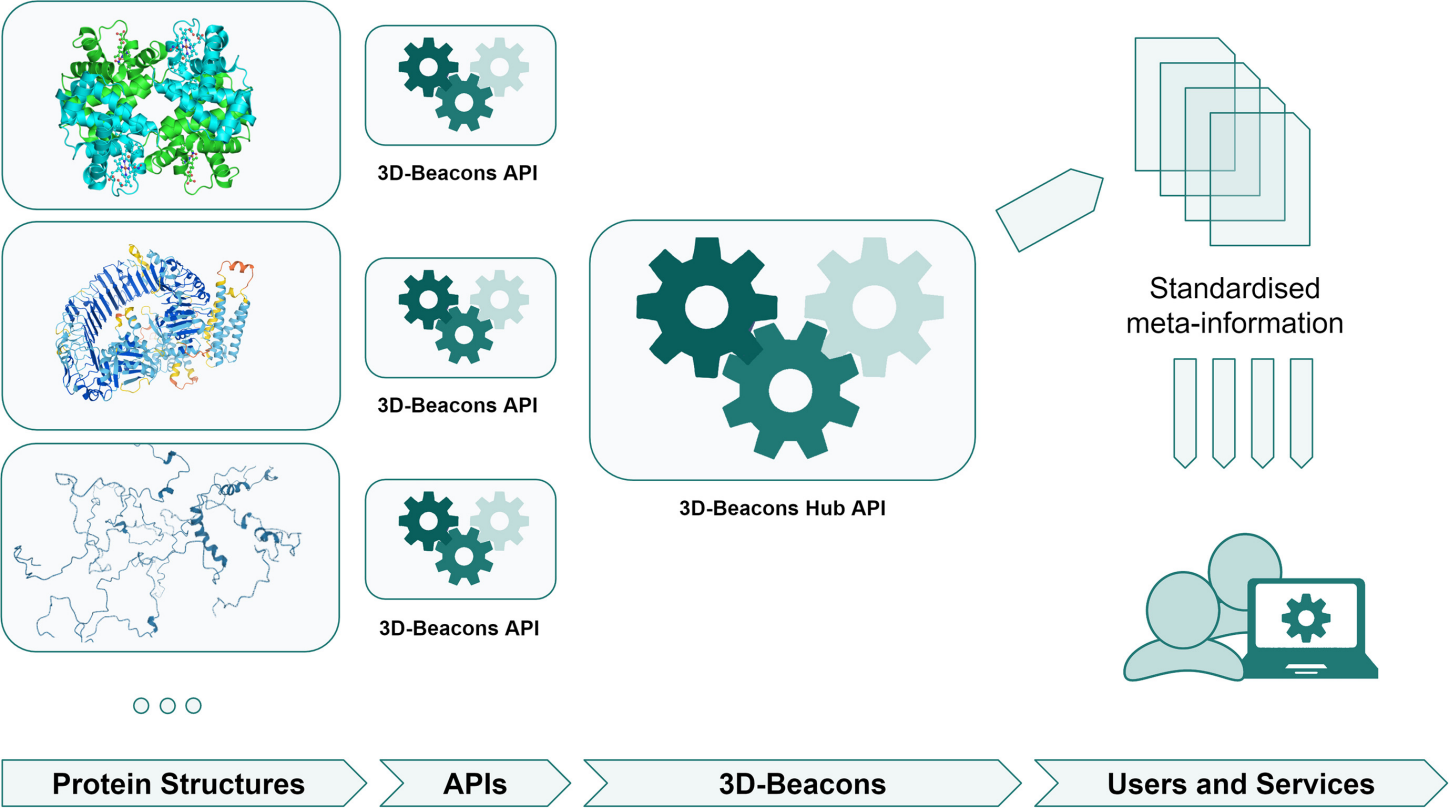
Schematic overview of the 3D-Beacons Network. Data providers standardize their meta-information and make their models available through 3D-Beacons API instances. The 3D-Beacons Registry links these instances to the central 3D-Beacons Hub API, which can be openly accessed by the scientific community and other data services.

The 3D-Beacons Network is based on an infrastructure that helps providers of protein structures to standardize their meta-information and easily link their model files to a centralized search engine, called the 3D-Beacons Hub API (application programming interface) (Fig. 3). Each data provider has its 3D-Beacon connected to the central hub. The hub is the public access point through which the users (or other data services) can retrieve models from any members. This allows users to get all structures for a given UniProt accession instead of manually retrieving them from all the different structure providers.

Thanks to the standardized data formats, the infrastructure ensures complete transparency in data provenance and allows users to easily compare protein structures and their relevant meta-information. This initiative has evolved in parallel with efforts to improve the standardization of the coordinate files for theoretical models. In particular, members of the 3D-Beacons Network contributed to the ModelCIF extension of the PDBx/mmCIF format, which supports more exhaustive meta-information and includes mappings to the corresponding UniProt accessions next to the atomic coordinates.

**Figure 4:**
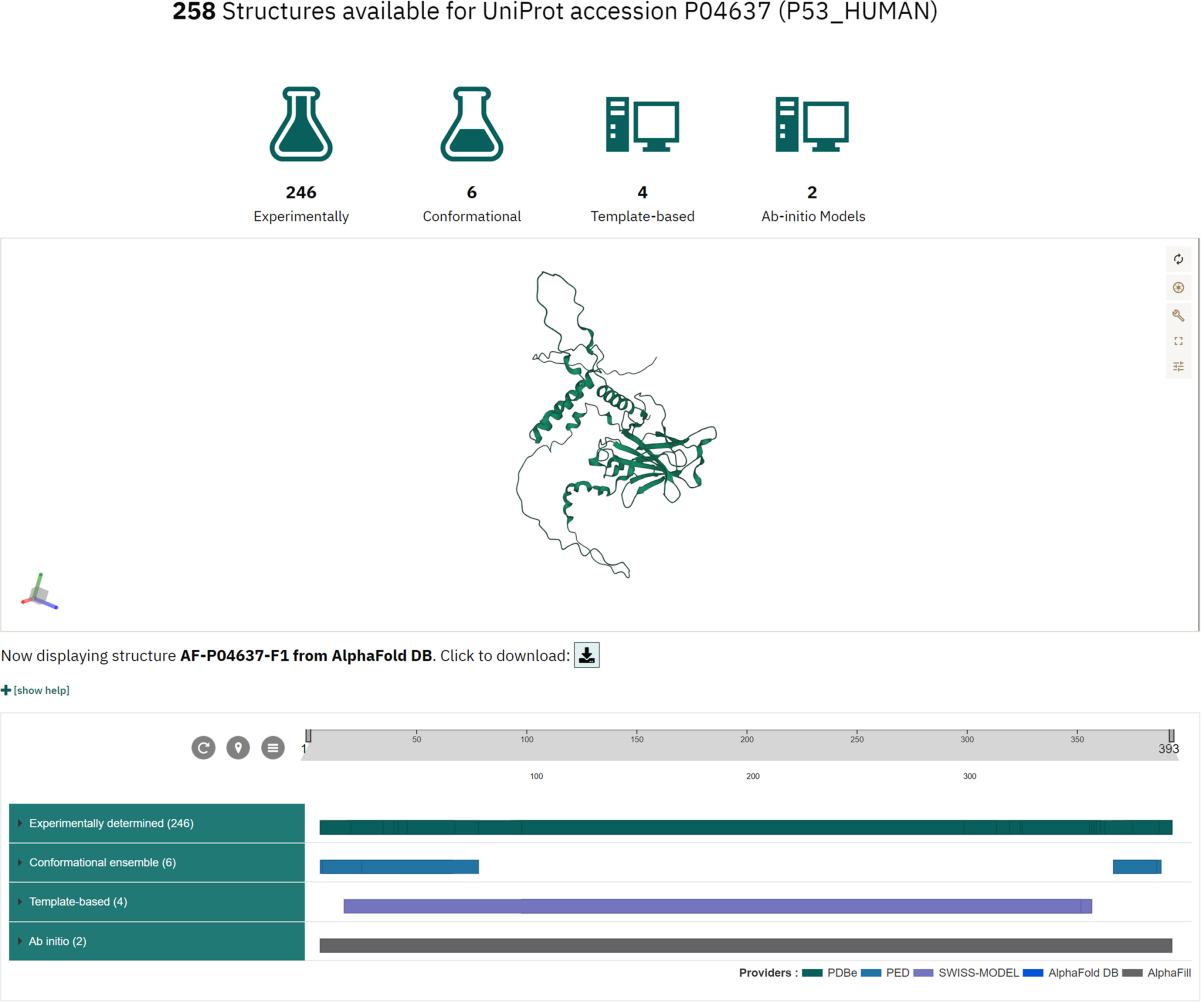
Graphical user interface of 3D-Beacons. While the main focus of the 3D-Beacons Network is to provide programmatic access to experimentally determined and computationally predicted protein structures, we also provide a graphical user interface where researchers can query for specific proteins using UniProt accessions. This interface displays which section of the protein sequence the models cover and provides an interactive 3-dimensional view.

While the primary purpose of 3D-Beacons is to provide efficient and scalable programmatic access to protein structures, we also offer a graphical user interface that allows researchers to get an overview of the available protein structures. For example, users can view all the available data from any member data provider for the human cellular tumor antigen p53 protein by searching based on its UniProt accession (Fig. 4).

We divided the protein structures into 4 categories: (i) experimentally determined, (ii) template based, (iii) *ab initio*, and (iv) conformational ensembles. We defined the categories as follows:

**Experimentally determined** structures are based on data from techniques such as X-ray crystallography, cryo-electron microscopy, nuclear magnetic resonance spectroscopy, or small-angle scattering. This category is exemplified by structures in the PDB and the SASBDB databases.

**Template-based** models use alignments to similar sequences with known structure (i.e., templates) as their main input. SWISS-MODEL is an example of data providers with such models.

***Ab initio*** models can use templates as an auxiliary input but do not depend on them. AlphaFold models are considered *ab initio* in this framework.

Finally, **conformational ensembles** are created using a combination of experimental data and computational modeling, yielding a large number of possible conformations. Ensembles in the PED database are an example of this category.

Researchers can view the number of models under each category and inspect which parts of the amino acid sequences are covered by which models in a 2D viewer, PDB ProtVista [30]. Users can also display the structures using an embedded 3-dimensional molecular graphics viewer, Mol* [31], and download the models in PDB or mmCIF formats.

## Discussion

The purpose of the 3D-Beacons Network is to standardize the representation of protein structure models and associated metadata and to provide efficient, high-throughput programmatic access to experimentally determined and theoretical models and their standardized metadata. The current version (as of 29 July 2022) of 3D-Beacons supports querying any number of UniProt accessions, while future updates are planned to collate models based on other identifiers such as taxonomy IDs or domain IDs. This platform enables both the scientific community and developers of data visualization and data-providing services to access and seamlessly integrate 3-dimensional models from various protein structure data providers.

While designing the data access points and data formats, we had extensive discussions with scientists and developers who provided specific use cases that are relevant to their work. We used these data to drive the development of 3D-Beacons, starting with the most frequently requested data (i.e., information keyed on UniProt accessions) that can answer the question, “What experimental or theoretical structures are available for my protein of interest?” Going forward, we will address more of the collated use cases, such as searching by sequence or by gene identifiers and selecting structures based on protein families. Already, the API endpoints of 3D-Beacons provide easy access to models from sparse and fragmented data resources, supporting researchers and software developers alike.

For example, the 3D-Beacons infrastructure allows users of Jalview, a workbench for creating multiple sequence alignments (MSAs) and analyzing them, to discover 3-dimensional models for MSAs of proteins from the UniProt and place them in the context of genetic variation from Ensembl [32]. It can also visualize local model quality scores such as pLDDT (Predicted Local Distance Difference Test).

The Protein Data Bank in Europe–Knowledge Base (PDBe-KB) [33] displays all the experimentally determined and computationally predicted structures for proteins of interest on their aggregated views of proteins. To retrieve metadata and the location of model files, it uses the 3D-Beacons Hub API. This integration also allows PDBe-KB to display functional and biophysical annotations both for theoretical models in addition to experimentally determined structures.

The SWISS-MODEL Repository (SMR) [13] fetches models from AlphaFold DB and the ModelArchive using the 3D-Beacons Hub API. SMR displays these models next to homology models from SWISS-MODEL [14] and experimental structures from the PDB [6] to facilitate comparative analysis. SMR also takes advantage of the confidence measure information, and the models are displayed with a consistent coloring based on these confidence metrics.

By providing easy access to experimentally determined and computationally predicted protein structures, we aim to make these data an essential part of the toolbox of researchers in the broader scientific fields of life sciences. Establishing an infrastructure of federated model providers if a scalable and expandable approach can efficiently adjust to include new models and provides a more sustainable model than if a single data repository would try and archive all the data in one place. By taking advantage of the 3D-Beacons Network, protein structures can better realize their full impact on fields from structure-based drug discovery [2, 34] to structural bioinformatics [35, 36] and from scientific software development [37] to experimental structure determination [38, 39]. The amount of available protein structures has never been as large as it is now, and providing convenient access to these models is a key service that will enable further research.

## Methods

The infrastructure of the 3D-Beacons Network consists of a registry, a hub, and the data access implementations. The 3D-Beacons Network is open to data providers of protein structures. Such data resources are invited to contact the 3D-Beacons consortium to discuss ways their data can be linked. Briefly, the common steps are as follows: data providers review the consortium guidelines and the latest API specification. The data providers then convert their metadata to the specified format and make these data available either through their APIs or by setting up a 3D-Beacon client. Once these steps are completed, the registry can be updated to link the new data resource with the 3D-Beacons Hub API. The following sections give more detailed information on each of these elements of the infrastructure.

### 3D-Beacons Registry

The 3D-Beacons Registry is a transparent, publicly accessible registry that stores information on all the data providers linked to the 3D-Beacons Network. The registry is available on GitHub. It contains information on the public URLs of data providers, a brief description of the protein structures they provide, and a list of API endpoints they support. For example, PDBe [40, 41] supports the API endpoint that is keyed on a UniProt accession and that provides high-level information about the models, while SMR [13] supports both the high-level and the detailed API endpoints, which additionally provides per-chain and per-residue information on the models.

### 3D-Beacons data exchange format

The API endpoints comply with the data exchange format, which the 3D-Beacons members collaboratively design and improve. We defined the data exchange format as a JavaScript Object Notation (JSON) specification, an industry-standard format for sharing textual meta-information. The specification is available on Apiary and GitHub.

### 3D-Beacons client

Members of the 3D-Beacons Network can either implement their own API endpoints according to the API specification described above or install a local instance of the 3D-Beacons client. This client is a Docker-containerized, lightweight Python package that can import and parse PDB or mmCIF formatted protein structure files and their corresponding meta-information (in JSON format). It also includes capabilities to add model confidence scores using QMEANDisCo [42] if models do not already include comparable scores such as pLDDT. QMEANDisCo, which is used internally by SWISS-MODEL, can be applied to models from any provider and has proven to be an accurate confidence predictor for homology modeling and some *ab initio* methods [20]. The client indexes the collated data in an embedded MongoDB database instance and exposes the information through an embedded API implementation that complies with the 3D-Beacons API specifications. The client is freely available on GitHub.

### 3D-Beacons Hub API

At the core of the 3D-Beacons infrastructure lies the Hub API, a programmatic aggregator of the meta-information from all the member data providers. We implemented the Hub API using the FastAPI framework. This API relies on the previously described registry to retrieve information on which data provider supports which specific API endpoints. It aggregates data and provides its own API endpoints that researchers, services, and software can directly access to retrieve the location of available model files and their corresponding meta-information, such as the overall model quality or residue-level confidence measures. It is important to note that in the current implementation, the model confidence measures are provided by the original data sources, and different providers might have different approaches to estimating confidence. This can hamper effective comparison of the models based on these scores, and it is an active focus area both within the 3D-Beacons Network and the broader modeling community to design a broadly applicable confidence measure.

### 3D-Beacons front-end

Finally, we provide a graphical user interface that contains documentation and showcases the information one can retrieve using the 3D-Beacons Hub API. We implemented this interface using the Angular framework, and it relies on the sequence feature viewer, PDB ProtVista [30], and the 3D molecular graphics viewer, Mol* [31]. The source code of this front-end application is available from GitHub.

## Availability of Supporting Source Code and Requirements

The source codes of the 3D-Beacons Registry, Client, Hub API, and front-end application are all publicly available:

Project name: 3D-Beacons

Project homepage: https://3d-beacons.org

Operating system(s): Platform independent

Programming language: Python, TypeScript

Other requirements: Python 3.7 or higher, Angular 11.1.3 or higher

License: Apache License 2.0

biotools: 3d-beacons

## Data Availability

All the data provided through the network are freely available for academic and commercial use under Creative Commons Attribution 4.0 license terms. Documentation of the 3D-Beacons Hub API is available at https://www.ebi.ac.uk/pdbe/pdbe-kb/3dbeacons/api/. The specification of the data exchange format is available at https://3dbeacons.docs.apiary.io/#. An archival copy of the code and other supporting data are also available via the *GigaScience* database GigaDB [43].

## Abbreviations

API: Application Programming Interface; IDP: intrinsically disordered proteins; JSON: JavaScript Object Notation; lDDT: local distance difference test; MSA: multiple sequence alignment; PDB: Protein Data Bank; PDBe-KB: Protein Data Bank in Europe–Knowledge Base; PED: Protein Ensemble Database; SASBDB: Small-Angle Scattering Biological Data Bank; SMR: SWISS-MODEL Repository; UniProt: Universal Protein Resource.

## Competing Interests

The authors declare no competing interests.

## Authors’ Contributions

M.V. created the initial draft, handled project and data management at PDBe and AlphaFold DB, and designed and developed the 3D-Beacons webpages. S.N. led the development of the 3D-Beacons registry, Hub API, and PDBe and AlphaFold API implementation, as well as contributed to the development of the 3D-Beacons client and the webpages. I.S. worked on the 3D-Beacons client and on the Genome3D beacon. G.T. contributed to the management of 3D-Beacons. M.V., G.T., A.M.W., and S.B. contributed to the API design. A.L. and N.A. provided management support for AlphaFold DB. A.H., S.T., L.T.-K., E.S., and D.P. contributed to the PED beacon. A.M.W., S.B., and G.T. contributed to the SWISS-MODEL and ModelArchive beacons. S.B. contributed QMEANDisCo for the client. S.B. and G.T. connected with the ModelCIF working group. T.H. and E.S. contributed to the HegeLab beacon. M.L.H. and R.P.J. contributed to the AlphaFill beacon. C.B., Dm.S, and D.M. contributed to the SASBDB beacon. M.S., M.J.S., and S.L.S. contributed to the isoform.io beacon. M.D. and S.A. worked on data visualization and infrastructure. S.V., C.O., and T.S. provided oversight as co-principal investigators. I.S., G.T., D.M., T.H., S.V., R.J., and E.S. contributed to the manuscript drafts. Every coauthor reviewed the final manuscript.

## Supplementary Material

giac118_GIGA-D-22-00215_Original_Submission

giac118_GIGA-D-22-00215_Revision_1

giac118_GIGA-D-22-00215_Revision_2

giac118_GIGA-D-22-00215_Revision_3

giac118_GIGA-D-22-00215_Revision_4

giac118_Response_to_Reviewer_Comments_Original_Submission

giac118_Response_to_Reviewer_Comments_Revision_1

giac118_Response_to_Reviewer_Comments_Revision_2

giac118_Response_to_Reviewer_Comments_Revision_3

giac118_Reviewer_1_Report_Original_Submission Lim Heo, Ph.D. -- 9/13/2022 Reviewed

giac118_Reviewer_2_Report_Original_Submission Carlos Rodrigues, PhD -- 9/16/2022 Reviewed
