# Supplementary material for: 3D-Beacons: decreasing the gap between protein sequences and structures through a federated network of protein structure data resources
Source: Gigascience. 2022 Nov 30;11:giac118. doi: 10.1093/gigascience/giac118 (PMC9709962; doi:10.1093/gigascience/giac118)

GigaScience

3D-Beacons: Decreasing the gap between protein sequences and structures through a federated network of protein structure data resources

--Manuscript Draft--

**Manuscript Number:**	GIGA-D-22-00215R1
**Full Title:**	3D-Beacons: Decreasing the gap between protein sequences and structures through a federated network of protein structure data resources
**Article Type:**	Technical Note
**Funding Information:**	Biotechnology and Biological Sciences Research Council (BB/S020071/1) — Dr Sameer Velankar<br>Bundesministerium für Forschung und Technologie (16QK10A-SAS-BSOFT) — Dr Dmitri Svergun
**Abstract:**	While scientists can often infer the biological function of proteins from their 3-dimensional quaternary structures, the gap between the number of known protein sequences and their experimentally determined structures keeps increasing. A potential solution to this problem is presented by ever more sophisticated computational protein modelling approaches. While often powerful on their own, most methods have strengths and weaknesses. Therefore, it benefits researchers to examine models from various model providers and perform comparative analysis to identify what models can best address their specific use cases.<br>To make data from a large array of model providers more easily accessible to the broader scientific community, we established 3D-Beacons, a collaborative initiative to create a federated network with unified data access mechanisms. The 3D-Beacons Network allows researchers to collate coordinate files and metadata for experimentally determined and theoretical protein models from state-of-the-art and specialist model providers and also from the Protein Data Bank.
**Corresponding Author:**	Mihaly Varadi<br>EMBL-EBI: European Bioinformatics Institute<br>Hinxton, UNITED KINGDOM
**Corresponding Author Secondary Information:**	
**Corresponding Author's Institution:**	EMBL-EBI: European Bioinformatics Institute
**Corresponding Author's Secondary Institution:**	
**First Author:**	Mihaly Varadi
**First Author Secondary Information:**	
**Order of Authors:**	Mihaly Varadi
	Sreenath Nair
	Ian Sillitoe
	Gerardo Tauriello
	Stephen Anyango
	Stefan Bienert
	Clemente Borges
	Mandar Deshpande
	Tim Green
	Andras Hatos
	Tamas Hegedus

	Maarten L Hekkelman
	Robbie Joosten
	John Jumper
	Agata Laydon
	Dmitry Molodenskiy
	Damiano Piovesan
	Edoardo Salladini
	Steven L Salzberg
	Markus J Sommer
	Martin Steinegger
	Erzsebet Suhajda
	Dmitri Svergun
	Luiggi Tenorio-Ku
	Silvio Tosatto
	Kathryn Tunyasuvunakool
	Andrew M Waterhouse
	Augustin Zidek
	Demis Hassabis
	Torsten Schwede
	Christine Orengo
	Sameer Velankar
**Order of Authors Secondary Information:**	
**Response to Reviewers:**	We thank the reviewers for their positive feedback and their constructive suggestions! Please find our responses below.<br><br>Review 1<br><br>Q1<br>1. I recently noticed that RCSB PDB also made it possible to search computational protein models by extending its web interface. The database included ~1 million models from AlphaFold DB and ~1,100 models from ModelArchive, which are main sources of this work as well and are maintained by some of the authors of this work. Even though the number of models and the diversity of the sources accessible via the RCSB PDB interface are fewer than this work, I think the purpose of both works are similar. As there are some overlaps between this work and the RCSB PDB interface in terms of data providers (and authors), what is the significance of this work compared to the RCSB PDB interface?<br><br>A1<br>Thank you for raising this question; we agree it is important to clarify the differences. The recent update in RCSB PDB is based on copying public data and indexing a subset of the data from AlphaFold DB and the ModelArchive. This allowed the developers to embed these models into the RCSB PDB search system. This approach has limitations, such as the lack of transparent data update mechanisms, providing access to incomplete data (i.e. no access to certain confidence metrics) and offering only subsets of data from a subset of data providers. In our approach, we worked together with AlphaFold DB, ModelArchive and other model providers to collaboratively design a federated network that we felt offers a more sustainable and transparent solution to the problem of providing open access to protein structure models. Through 3D-Beacons, all the data providers make all their data accessible, as 3D-Beacons

Powered by Editorial Manager® and ProduXion Manager® from Aries Systems Corporation

doesn't depend on copying data but rather ensures the data providers can link their own, up-to-date datasets to the network. We added a few sentences to provide similar justification as above.

Q2
2. Most computational models rely on a few data providers, AlphaFold DB, SWISS-MODEL Repository, and AlphaFill (for ligands). In my opinion, it would be better to make the platform richer by recruiting more diverse data providers with different points of view (e.g., conformational ensembles) or different modeling approaches (e.g., machine learning-based approaches with pre-trained protein language models such as OmegaFold). Is there any plan for such progress or promotion of the platform?

A2
Indeed, we envision 3D-Beacons to be an open platform for any data provider can join who agrees to the collaboration guidelines (https://www.ebi.ac.uk/pdbe/pdbe-kb/3dbeacons/guidelines). We have conformational ensembles from the Protein Ensemble Database and SASBDB, but we would welcome more data providers, from small research teams with high quality structures to large datasets from major data resources. Another example of structures we are keen to link, and are in the progress of doing so, is mutant structures from resources such as Missense3D and FoldX. We added a few sentences to make our intentions clearer.

Q3
3. It would be better to have a guide of model selection if there are multiple searched models for an Uniprot ID. Alternatively, providing universal quality assessment scores for models would be an option (by additional data provider). Currently, pLDDT scores are provided, but they are difficult to compare between modeling methods as they were trained independently for each method.

A3
We thank the reviewer for this comment; we are working together with model providers to try and come up with a confidence metric that would allow meaningful ranking of predicted protein structures. We are working on including QMEANDisco in the 3D-Beacons client to provide an assessment tool to data providers who might lack a confidence measure, but this is only a step towards having a unified quality metric we could use to sort/rank the model structures. We added a few sentences to explain this limitation and mention that it is an ongoing effort.

Q4
4. I was able to search on the 3D-Beacons web page a few days ago. However, I could not at the moment of writing these review comments (Sept. 13, 6 p.m. in EDT).

A4
Thank you for reporting this! We will check the traffic logs to see what might have caused the intermittent failure. There is a large-scale compute centre migration happening at EMBL-EBI that affects every EBI-hosted service, which might have caused this temporary outage.

Review 2:

Q5
A minor correction is required on page 7, where the authors describe 4 different types of protein structures: Experimentally determined, Template-based, Ab-initio anc Conformational Ensembles. On many examples available on the website (e.g. https://www.ebi.ac.uk/pdbe/pdbe-kb/3dbeacons/search/P15056), there is one extra category which is structures derived from "Deep learning" methods. I am assuming this comprises a sub-set of Ab-initio structures, which the authors decided to keep as a separate category after submitting this study for publication. The main text should be updated to reflect this change as well as Figure 4.

A5
Thank you very much for raising this inconsistency! This was a bug which we now fixed - There was a major change in the underlying API which included the merging of the

	categories. This particular update was not yet deployed on the production server - We fixed this, and now the correct categories are visible in the live web pages.
**Additional Information:**	
**Question**	**Response**
Are you submitting this manuscript to a special series or article collection?	No
**Experimental design and statistics**<br><br>Full details of the experimental design and statistical methods used should be given in the Methods section, as detailed in our Minimum Standards Reporting Checklist. Information essential to interpreting the data presented should be made available in the figure legends.<br><br>Have you included all the information requested in your manuscript?	Yes
**Resources**<br><br>A description of all resources used, including antibodies, cell lines, animals and software tools, with enough information to allow them to be uniquely identified, should be included in the Methods section. Authors are strongly encouraged to cite Research Resource Identifiers (RRIDs) for antibodies, model organisms and tools, where possible.<br><br>Have you included the information requested as detailed in our Minimum Standards Reporting Checklist?	Yes
**Availability of data and materials**<br><br>All datasets and code on which the conclusions of the paper rely must be either included in your submission or deposited in publicly available repositories (where available and ethically appropriate), referencing such data using a unique identifier in the references and in the “Availability of Data and Materials”	Yes

section of your manuscript.<br><br>Have you have met the above requirement as detailed in our Minimum Standards Reporting Checklist?	

Manuscript

Click here to access/download;Manuscript;3D-Beacons manuscript - Revised - GigaScience.docx

3D-Beacons: Decreasing the gap between protein sequences and structures through a federated network of protein structure data resources

Mihaly Varadi[1+], Sreenath Nair[1+], Ian Sillitoe[2+], Gerardo Tauriello[3,12+], Stephen Anyango[1], Stefan Bienert[3,12], Clemente Borges[4], Mandar Deshpande[1], Tim Green[5], Demis Hassabis[5], Andras Hatos[6,7], Tamas Hegedus[8], Maarten L Hekkelman[9], Robbie Joosten[9], John Jumper[5], Agata Laydon[5], Dmitry Molodenskiy[4], Damiano Piovesan[6], Edoardo Salladini[6], Steven L. Salzberg[10], Markus J Sommer[10], Martin Steinegger[11], Erzsebet Suhajda[8], Dmitri Svergun[4], Luiggi Tenorio-Ku[6], Silvio Tosatto[6], Kathryn Tunyasuvunakool[5], Andrew Mark Waterhouse[3,12], Augustin Žídek[5], Torsten Schwede[3,12]*, Christine Orengo[2]*, Sameer Velankar[1]*

1 European Molecular Biology Laboratory, European Bioinformatics Institute, Hinxton, UK
2 UCL, Department of Structural and Molecular Biology, London, UK
3 University of Basel, Biozentrum, Basel, Switzerland
4 European Molecular Biology Laboratory, EMBL Hamburg, Hamburg, Germany
5 DeepMind, London, UK
6 University of Padova, Department of Biomedical Sciences, Padova, Italy
7 University of Lausanne, SIB, Lausanne, Switzerland
8 Semmelweis University, Department of Biophysics and Radiation Biology, Budapest, Hungary
9 Netherlands Cancer Institute, Amsterdam, The Netherlands
10 Johns Hopkins University, Biomedical Engineering, Baltimore, MD, USA
11 Seoul National University, School of Biology, Seoul, South Korea
12 SIB Swiss Institute of Bioinformatics, Computational Structural Biology, Basel, Switzerland

+ these authors contributed equally

* corresponding authors

Abstract

While scientists can often infer the biological function of proteins from their 3-dimensional quaternary structures, the gap between the number of known protein sequences and their experimentally determined structures keeps increasing. A potential solution to this problem is presented by ever more sophisticated computational protein modelling approaches. While often powerful on their own, most methods have strengths and weaknesses. Therefore, it benefits researchers to examine models from various model providers and perform comparative analysis to identify what models can best address their specific use cases.

To make data from a large array of model providers more easily accessible to the broader scientific community, we established 3D-Beacons, a collaborative initiative to create a federated network with unified data access mechanisms. The 3D-Beacons Network allows researchers to collate coordinate files and metadata for experimentally determined and theoretical protein models from state-of-the-art and specialist model providers and also from the Protein Data Bank.

Introduction

Proteins are essential building blocks of almost every biological process; therefore, understanding their functions is critical to many applications, from drug discovery[1,2] to tackling environmental challenges such as plastic pollution[3]. Accurate information on the structure of a protein, especially in the context of its biological assembly, can help scientists understand and modulate its function[4,5].

Unfortunately, gaining such insights regarding the function of proteins through their structures is severely hampered by the lack of high-quality, experimentally determined structures. As of 2022, the Universal Protein Resource (UniProt) contains around 204 million non-redundant amino acid sequences, while the Protein Data Bank (PDB)[6,7] contains around 190,000 PDB entries mapped to approximately 52,000 UniProt accessions. In other words, less than 0.03% of all the known protein sequences have experimentally determined atomic resolution structures. As sequencing becomes more accessible, the gap between protein sequences and structures increases (Figure 1).

A practical approach to addressing this challenge relies on high-accuracy computational models to complement the experimentally determined structures when the latter are unavailable for a certain protein of interest[8]. The thermodynamic hypothesis postulates that within certain limitations, the native structure is determined only by the protein's amino acid sequence[9,10]. Indeed, the past 50 years saw the development of many algorithms and scientific software to predict protein structures[11,12]. An approach developed early in this field was to use homologous protein structures as templates. Several modelling tools and data resources have long provided access to such models, for example, the SWISS-MODEL and the ModBase web-services and databases[13–15]. In 2021 the field saw tremendous advances with tools such as AlphaFold and RoseTTAFold achieving much higher accuracy for "*de novo*" predictions without homologous templates than ever before[16,17]. This new generation of prediction tools makes it possible to try and predict the structure of virtually any known protein based on its sequence.

While these new techniques are increasingly accurate, it is important that they are supplemented with reliable estimates of model confidence both for the whole model and locally for each residue. Researchers should not expect all predictions to be equally accurate neither globally nor in every region, and confidence estimates should hence be used to determine if a predicted structure can be used for downstream analysis[18]. Commonly used model confidence methods aim to predict the global and local similarity of the model compared to the correct coordinates if those coordinates were provided by an experimentally determined structure. In recent years, several model prediction methods such as SWISS-MODEL[14], RoseTTAFold[17] and AlphaFold[16] have chosen the superposition-free lDDT score[19] as a similarity metric to provide model confidence for their own models. The lDDT score measures differences in interatomic distances within a short radius between model and reference structure. It has been shown that superposition-free measures are robust with respect to domain movements and have advantages for the analysis of local structural details[20]. Similarly, superposition-free measures have been used for a long time in the creation of experimental structure models[21].

Another important consideration when relying on any structure prediction tool is to consider its limitations. While structures in the PDB have the advantage of experimental data backing the coordinates, enabling experimental as well as geometric validation, it is a relatively small data set, as discussed above. Template-based models have the distinct advantage of enabling the mapping of a model to homologues with known structures, thus mapping to experimentally derived structures which can be in distinct conformational states or in complex with other molecules. Some tools excel at general-purpose protein structure modelling; others specialise in placing relevant ligands in the context of a model or representing conformational flexibility with ensembles of potential conformations[14,16,22–24] (Figure 2). For example, AlphaFold 2.0 cannot perform docking of small molecules, even if they are obligate ligands of the proteins, such as Zinc-finger proteins. However, data resources such as AlphaFill can tackle this problem by building on existing models and adding known ligands to these structures[23] (Figure 2A). On the other hand, the central repository of AlphaFold models, the AlphaFold Structure Database, only contains predictions for single polypeptide chains and not necessarily the functional forms of proteins[25]. In the case of multimeric complexes, the functional form can include several polypeptide chains. Since the number of known protein complexes is immense, having a comprehensive database for complex structures soon is rather unlikely. Therefore, integrating 3D data from experts in specialised fields of proteins is important, as demonstrated by physiologically and pathologically relevant transmembrane ABC half transporters[26] and by a set of computed structures of core eukaryotic protein complexes deposited in the ModelArchive[27]. Databases

such as the Small-Angle Scattering Biological Data Bank (SASBDB)[28] and the Protein Ensemble Database (PED)[22] highlight the dynamic nature of intrinsically disordered proteins (Figure 2B). Small-angle scattering provides low-resolution information on the shape and size of biological macromolecules in solution, but it also offers powerful means for the quantitative analysis of flexible systems, including intrinsically disordered proteins (IDPs)[29]. This data together with *ab initio* modelling approaches can be utilised to generate an experimentally validated pool of IDP models. PED provides access to such conformational ensembles, but also those based on other experimental approaches. Considering the limitations of certain tools highlights the importance of using models and methods from various synergistic software and data providers to mitigate the weaknesses of individual modelling techniques.

While many prediction software and several publicly accessible data resources host and archive protein structures, these resources are fragmented and often rely on their own data standards to describe the necessary meta-information essential for providing context for a specific model. They also offer distinct data access mechanisms, requiring the users to learn multiple sets of technical details when interacting with various resources. The lack of standardisation can severely impede the comparative analysis of these models, making it difficult to gain valuable insights.

Here, we present the 3D-Beacons Network (https://3d-beacons.org), an open, collaborative platform for providing programmatic access to 3-dimensional coordinates and their standardised meta-information from both experimentally determined and computationally modelled protein structures.

Results

The 3D-Beacons Network is an open collaboration between providers of experimentally determined and computationally predicted protein structures. To date, ten data providers make their protein structures available through this platform (Table 1). The consortium is guided by a collaboration agreement that prospective data providers agree to comply with (https://www.ebi.ac.uk/pdbe/pdbe-kb/3dbeacons/guidelines). We encourage and invite macromolecular structure providers from research teams focusing on small, curated datasets to large data resources to join the 3D-Beacons Network and take advantage of its infrastructure to make their models more accessible to the scientific community. Importantly, all the data provided through the network must be freely available for academic and

commercial use under Creative Commons Attribution 4.0 (CC-BY 4.0, https://creativecommons.org/licenses/by/4.0/) licence terms.

The 3D-Beacons Network is based on an infrastructure that helps providers of protein structures to standardise their meta-information, and easily link their model files to a centralised search engine, called the 3D-Beacons Hub API (application programming interface) (Figure 3). Each data provider has its 3D-Beacon connected to the central Hub. The Hub is the public access point through which the users (or other data services) can retrieve models from any members. This allows users to get all structures for a given UniProt accession instead of manually retrieving them from all the different structure providers.

Thanks to the standardised data formats, the infrastructure assures complete transparency in data provenance and allows users to easily compare protein structures and their relevant meta-information. This initiative has evolved in parallel with efforts to improve the standardisation of the coordinate files for theoretical models. In particular, members of the 3D-Beacons Network contributed to the ModelCIF extension of the PDBx/mmCIF format, which supports more exhaustive meta-information and includes mappings to the corresponding UniProt accessions next to the atomic coordinates.

While the primary purpose of 3D-Beacons is to provide efficient and scalable programmatic access to protein structures, we also offer a graphical user interface that allows researchers to get an overview of the available protein structures. For example, users can view all the available data from any member data provider for the human Cellular tumour antigen p53 protein by searching based on its UniProt accession: https://www.ebi.ac.uk/pdbe/pdbe-kb/3dbeacons/search/P04637 (Figure 4).

We divided the protein structures into four categories: 1) experimentally determined; 2) template-based; 3) *ab-initio*; and 4) conformational ensembles. We defined the categories as follows:

Experimentally determined structures are based on data from techniques such as X-ray crystallography, cryo-electron microscopy, nuclear magnetic resonance spectroscopy or small-angle scattering. This category is exemplified by structures in the PDB and the SASBDB databases.

Template-based models use alignments to similar sequences with known structure (i.e. templates) as their main input. SWISS-MODEL is an example of data providers with such models.

Ab-initio models can use templates as an auxiliary input, but do not depend on them. AlphaFold models are considered *ab-initio* in this framework.

Finally, **conformational ensembles** are created using a combination of experimental data and computational modelling, yielding a large number of possible conformations. Ensembles in the PED database are an example of this category.

Researchers can view the number of models under each category and inspect which parts of the amino acid sequences are covered by which models in a 2D viewer, PDB ProtVista[30]. Users can also display the structures using an embedded 3D molecular graphics viewer, Mol*[31], and download the models in PDB or mmCIF formats.

Discussion

The purpose of the 3D-Beacons Network is to standardise the representation of protein structure models and associated metadata and to provide efficient, high-throughput programmatic access to experimentally determined and theoretical models and their standardised metadata. The current version (as of 29th July 2022) of 3D-Beacons supports querying any number of UniProt accessions, while future updates are planned to collate models based on other identifiers such as taxonomy IDs or domain IDs. This platform enables both the scientific community and developers of data visualisation and data providing services to access and seamlessly integrate 3D models from various protein structure data providers.

While designing the data access points and data formats, we had extensive discussions with scientists and developers who provided specific use cases that are relevant to their work. We used this data to drive the development of 3D-Beacons, starting with the most frequently requested data, i.e. information keyed on UniProt accessions, that can answer the question “What experimental or theoretical structures are available for my protein of interest?”. Going forward, we will address more of the collated use cases, such as searching by sequence or by gene identifiers and selecting structures based on protein families. Already, the API endpoints of 3D-Beacons provide easy access to models from sparse and fragmented data resources, supporting researchers and software developers alike.

For example, the 3D-Beacons infrastructure allows users of Jalview, a workbench for creating multiple sequence alignments (MSAs) and analysing them, to discover 3D models for MSAs of proteins from the UniProt and place them in the context of genetic variation from Ensembl[32]. It can also visualise local model quality scores such as pLDDT.

The Protein Data Bank in Europe – Knowledge Base (PDBe-KB)[33] displays all the experimentally determined and computationally predicted structures for proteins of interest on their aggregated views of proteins. To retrieve metadata and the location of model files, it uses the 3D-Beacons Hub API. This integration also allows PDBe-KB to display functional and biophysical annotations both for theoretical models in addition to experimentally determined structures.

The SWISS-MODEL Repository (SMR)[13] fetches models from AlphaFold DB and the ModelArchive using the 3D-Beacons Hub API. SMR displays these models next to homology models from SWISS-MODEL[14] and experimental structures from the PDB[6] to facilitate comparative analysis. SMR also takes advantage of the confidence measure information, and the models are displayed with a consistent colouring based on these confidence metrics.

By providing easy access to experimentally determined and computationally predicted protein structures, we aim to make these data an essential part of the toolbox of researchers in the broader scientific fields of life sciences. Establishing an infrastructure of federated model providers if a scalable and expandable approach that can efficiently adjust to include new models and provides a more sustainable model than if a single data repository would try and archive all the data in one place. By taking advantage of the 3D-Beacons Network, protein structures can better realise their full impact on fields from structure-based drug discovery[2,34] to structural bioinformatics[35,36], and from scientific software development[37] to experimental structure determination[38,39]. The amount of available protein structures has never been as large as it is now, and providing convenient access to these models is a key service that will enable further research.

Methods

The infrastructure of the 3D-Beacons Network consists of a registry, a hub and the data access implementations. The 3D-Beacons Network is open to data providers of protein structures. Such data resources are invited to contact the 3D-Beacons consortium to discuss ways their data can be linked. Briefly, the common steps are as follows: Data providers review the consortium guidelines (https://www.ebi.ac.uk/pdbe/pdbe-kb/3dbeacons/guidelines) and the latest API specification.The data providers then convert their metadata to the specified format and make these data available either through their APIs or by setting up a 3D-Beacon client. Once these steps are completed, the registry can be updated to link the new data resource with the 3D-Beacons Hub API. The following

sections below give more detailed information on each of these elements of the infrastructure.

3D-Beacons Registry

The 3D-Beacons Registry is a transparent, publicly accessible registry that stores information on all the data providers linked to the 3D-Beacons Network. The registry is available on GitHub (https://github.com/3D-Beacons/3d-beacons-registry). It contains information on the public URLs of data providers, a brief description of the protein structures they provide, and a list of API endpoints they support. For example, PDBe[40,41] supports the API endpoint that is keyed on a UniProt accession, and that provides high-level information about the models, while SMR[13] supports both the high-level and the detailed API endpoints, which additionally provides per-chain and per-residue information on the models.

3D-Beacons data exchange format

The API endpoints comply with the data exchange format, which the 3D-Beacons members collaboratively design and improve. We defined the data exchange format as a JavaScript Object Notation (JSON) specification, an industry-standard format for sharing textual meta-information. The specification is available on Apiary (https://3dbeacons.docs.apiary.io/#) and GitHub (https://github.com/3D-Beacons/3d-beacons-specifications/releases).

3D-Beacons client

Members of the 3D-Beacons Network can either implement their own API endpoints according to the API specification described above, or they can install a local instance of the 3D-Beacons client. This client is a Docker-containerized, lightweight Python package that can import and parse PDB or mmCIF formatted protein structure files and their corresponding meta-information (in JSON format). It also includes capabilities to add model confidence scores using QMEANDisCo[42] if models do not already include comparable scores such as pLDDT. QMEANDisCo, which is used internally by SWISS-MODEL, can be applied to models from any provider and has proven to be an accurate confidence predictor for homology modelling and some *ab initio* methods[20]. The client indexes the collated data in an embedded MongoDB database instance and exposes the information through an embedded API implementation that complies with the 3D-Beacons API specifications. The client is freely available on GitHub (https://github.com/3D-Beacons/3d-beacons-client).

3D-Beacons Hub API

At the core of the 3D-Beacons infrastructure lies the Hub API, a programmatic aggregator of the meta-information from all the member data providers. We implemented the Hub API using the FastAPI framework. The source code of this API is available at https://github.com/3D-Beacons/3d-beacons-hub-api. This API relies on the previously described registry to retrieve information on which data provider supports which specific API endpoints. It aggregates data and provides its own API endpoints that researchers, services and software can directly access to retrieve the location of available model files and their corresponding meta-information, such as the overall model quality or residue-level confidence measures. It is important to note that in the current implementation the model confidence measures are provided by the original data sources, and different providers might have different approaches to estimating confidence. This can hamper effective comparison of the models based on these scores, and it is an active focus area both within the 3D-Beacons Network and the broader modelling community to design a broadly applicable confidence measure.

3D-Beacons front-end

Finally, we provide a graphical user interface that contains documentation and showcases the information one can retrieve using the 3D-Beacons Hub API at https://3d-beacons.org. We implemented this interface using the Angular framework, and it relies on the sequence feature viewer, PDB ProtVista[30] and the 3D molecular graphics viewer, Mol*[31]. The source code of this front-end application is available at https://github.com/3D-Beacons/3d-beacons-front-end.

Data availability

All the data provided through the network is freely available for academic and commercial use under Creative Commons Attribution 4.0 (CC-BY 4.0, https://creativecommons.org/licenses/by/4.0/) licence terms. Documentation of the 3D-Beacons Hub API is available at https://www.ebi.ac.uk/pdbe/pdbe-kb/3dbeacons/api/. The specification of the data exchange format is available at https://3dbeacons.docs.apiary.io/#. The source code of the 3D-Beacons Registry, Client, Hub API and front-end application are all publicly available at https://github.com/3D-Beacons.

Abbreviations

API: Application Programming Interface; IDP: Intrinsically Disordered Proteins; JSON: JavaScript Object Notation; lDDT: Local Distance Difference Test; MSA: Multiple Sequence Alignment; PDB: Protein Data Bank; PDBe-KB: Protein Data Bank in Europe - Knowledge Base; PED: Protein Ensemble Database; SASBDB: Small-Angle Scattering Biological Data Bank; SMR: SWISS-MODEL Repository; UniProt: Universal Protein Resource.

Figure legends

Figure 1 - Growth of the UniProt and the PDB databases
This figure shows the number of accessions (on a logarithmic scale) throughout the past decade. In 2011, the UniProt had 161× as many protein sequences as the number of PDB entries. This ratio grew by an order of magnitude, and was 1132 to 1 in 2021, showing that the gap between known protein sequences and their structures keeps increasing.

Figure 2 - Highlighting the strengths and weaknesses of modelling techniques
Each modelling approach has limitations and specific strengths. For example, AlphaFill complements AlphaFold models by placing obligate ligands in their contexts (panel A). Other data providers, such as the Protein Ensemble Database, provide conformational ensembles for intrinsically disordered proteins (IDPs), for example, for the human Alpha-synuclein (panel B).

Figure 3 - Schematic overview of the 3D-Beacons Network
Data providers standardise their meta-information and make their models available through 3D-Beacons API instances. The 3D-Beacons Registry links these instances to the central 3D-Beacons Hub API, which can be openly accessed by the scientific community and other data services.

Figure 4 - Graphical user interface of 3D-Beacons
While the main focus of the 3D-Beacons Network is to provide programmatic access to experimentally determined and computationally predicted protein structures, we also provide a graphical user interface where researchers can query for specific proteins using UniProt accessions. This interface displays which section of the protein sequence the models cover and provides an interactive 3D view.

Competing interests

The authors declare no competing interests.

Acknowledgements

Work on creating the 3D-Beacons infrastructure was primarily funded by the BBSRC grant BB/S020071/1. We would like to acknowledge the contribution of ELIXIR BioHackathon participants in 2020 and 2021 who helped improve various aspects of the 3D-Beacons infrastructure. G.T., A.M.W., S.B. and T.S. acknowledge funding from ELIXIR and the SIB Swiss Institute of Bioinformatics. D.I.S. and D.S.M. acknowledge funding from the German Ministry of Science and Education Grant Number: 16QK10A-SAS-BSOFT. M.S. acknowledges support from the National Research Foundation of Korea (NRF) Grant Number: 2021-R1C1-C102065.

Author’s contributions

M.V. created the initial draft, handled project and data management at PDBe and AlphaFold DB, and designed and developed the 3D-Beacons web pages. S.N. led the development of the 3D-Beacons registry, Hub API and PDBe and AlphaFold API implementation, as well as contributed to the development of the 3D-Beacons client and the web pages. I.S. worked on the 3D-Beacons client, and on the Genome3D beacon. G.T. contributed to the management of 3D-Beacons. M.V., G.T., A.M.W., S.B. contributed to the API design. A.L., N.A. provided management support for AlphaFold DB. A.H., S.T., L.T-K., E.S., D.P. contributed to the PED beacon. A.M.W., S.B., G.T. contributed to the SWISS-MODEL and ModelArchive beacons. S.B. contributed QMEANDisCo for the client. S.B., G.T. connected with the ModelCIF working group. T.H., E.S. contributed to the HegeLab beacon. M.L.H., R.P.J. contributed to the AlphaFill beacon. C.B., Dm.S, D.M. contributed to the SASBDB beacon. M.S., M.J.S., S.L.S. contributed to the isoform.io beacon. M.D., S.A. worked on data visualisation and infrastructure. S.V., C.O., T.S. provided oversight as co-PIs. I.S., G.T., D.M., T.H., S.V., R.J., E.S. contributed to the manuscript drafts. Every co-author reviewed the final manuscript.

Availability of supporting source code and requirements

Project name: 3D-Beacons

Project home page: https://github.com/3D-Beacons

Operating system(s): Platform independent

Programming language: Python, TypeScript

Other requirements: Python 3.7 or higher, Angular 11.1.3 or higher

License: Apache License 2.0

RRID: biotools:3d-beacons

References

1. Batool, M., Ahmad, B. & Choi, S. A Structure-Based Drug Discovery Paradigm. *Int. J. Mol. Sci.* **20**, 2783 (2019).
2. Ochoa, D. *et al.* Open Targets Platform: supporting systematic drug-target identification and prioritisation. *Nucleic Acids Res.* **49**, D1302–D1310 (2021).
3. Zhu, B., Wang, D. & Wei, N. Enzyme discovery and engineering for sustainable plastic recycling. *Trends Biotechnol.* **40**, 22–37 (2022).
4. Lee, D., Redfern, O. & Orengo, C. Predicting protein function from sequence and structure. *Nat. Rev. Mol. Cell Biol.* **8**, 995–1005 (2007).
5. PDBe-KB consortium *et al.* PDBe-KB: a community-driven resource for structural and functional annotations. *Nucleic Acids Res.* **48**, D344–D353 (2020).
6. wwPDB consortium *et al.* Protein Data Bank: the single global archive for 3D macromolecular structure data. *Nucleic Acids Res.* **47**, D520–D528 (2019).
7. UniProt Consortium. UniProt: the universal protein knowledgebase in 2021. *Nucleic Acids Res.* **49**, D480–D489 (2021).
8. Akdel, M. *et al. A structural biology community assessment of AlphaFold 2 applications*. http://biorxiv.org/lookup/doi/10.1101/2021.09.26.461876 (2021) doi:10.1101/2021.09.26.461876.
9. Anfinsen, C. B. Principles that govern the folding of protein chains. *Science* **181**, 223–230 (1973).
10. Hirata, F., Sugita, M., Yoshida, M. & Akasaka, K. Perspective: Structural fluctuation of protein and Anfinsen’s thermodynamic hypothesis. *J. Chem. Phys.* **148**, 020901 (2018).
11. Masrati, G. *et al.* Integrative Structural Biology in the Era of Accurate Structure Prediction. *J. Mol. Biol.* 167127 (2021) doi:10.1016/j.jmb.2021.167127.
12. Pereira, J. *et al.* High-accuracy protein structure prediction in CASP14. *Proteins* **89**, 1687–1699 (2021).
13. Bienert, S. *et al.* The SWISS-MODEL Repository-new features and functionality. *Nucleic*

Acids Res. **45**, D313–D319 (2017).

14. Waterhouse, A. *et al.* SWISS-MODEL: homology modelling of protein structures and complexes. *Nucleic Acids Res.* **46**, W296–W303 (2018).
15. Pieper, U. *et al.* ModBase, a database of annotated comparative protein structure models and associated resources. *Nucleic Acids Res.* **42**, D336-346 (2014).
16. Jumper, J. *et al.* Highly accurate protein structure prediction with AlphaFold. *Nature* (2021) doi:10.1038/s41586-021-03819-2.
17. Baek, M. *et al.* Accurate prediction of protein structures and interactions using a three-track neural network. *Science* **373**, 871–876 (2021).
18. Schwede, T. Protein Modeling: What Happened to the “Protein Structure Gap”? *Structure* **21**, 1531–1540 (2013).
19. Mariani, V., Biasini, M., Barbato, A. & Schwede, T. lDDT: a local superposition-free score for comparing protein structures and models using distance difference tests. *Bioinformatics* **29**, 2722–2728 (2013).
20. Olechnovič, K., Monastyrskyy, B., Kryshtafovych, A. & Venclovas, Č. Comparative analysis of methods for evaluation of protein models against native structures. *Bioinformatics* **35**, 937–944 (2019).
21. Smart, O. S. *et al.* Exploiting structure similarity in refinement: automated NCS and target-structure restraints in BUSTER. *Acta Crystallogr. D Biol. Crystallogr.* **68**, 368–380 (2012).
22. Lazar, T. *et al.* PED in 2021: a major update of the protein ensemble database for intrinsically disordered proteins. *Nucleic Acids Res.* **49**, D404–D411 (2021).
23. Hekkelman, M. L., de Vries, I., Joosten, R. P. & Perrakis, A. *AlphaFill: enriching the AlphaFold models with ligands and co-factors*. http://biorxiv.org/lookup/doi/10.1101/2021.11.26.470110 (2021) doi:10.1101/2021.11.26.470110.
24. Waman, V. P. *et al.* The Genome3D Consortium for Structural Annotations of Selected Model Organisms. *Methods Mol. Biol. Clifton NJ* **2165**, 27–67 (2020).

25. Varadi, M. *et al.* AlphaFold Protein Structure Database: massively expanding the structural coverage of protein-sequence space with high-accuracy models. *Nucleic Acids Res.* **50**, D439–D444 (2022).

26. Tordai, H. *et al. Comprehensive collection and prediction of ABC transmembrane protein structures in the AI era of structural biology*. http://biorxiv.org/lookup/doi/10.1101/2022.07.08.499254 (2022) doi:10.1101/2022.07.08.499254.

27. Humphreys, I. R. *et al.* Computed structures of core eukaryotic protein complexes. *Science* **374**, eabm4805 (2021).

28. Kikhney, A. G., Borges, C. R., Molodenskiy, D. S., Jeffries, C. M. & Svergun, D. I. SASBDB: Towards an automatically curated and validated repository for biological scattering data. *Protein Sci. Publ. Protein Soc.* **29**, 66–75 (2020).

29. Kikhney, A. G. & Svergun, D. I. A practical guide to small angle X-ray scattering (SAXS) of flexible and intrinsically disordered proteins. *FEBS Lett.* **589**, 2570–2577 (2015).

30. Deshpande, M. *et al. PDB ProtVista: A reusable and open-source sequence feature viewer*. http://biorxiv.org/lookup/doi/10.1101/2022.07.22.500790 (2022) doi:10.1101/2022.07.22.500790.

31. Sehnal, D. *et al.* Mol* Viewer: modern web app for 3D visualization and analysis of large biomolecular structures. *Nucleic Acids Res.* **49**, W431–W437 (2021).

32. Procter, J. B. *et al.* Alignment of Biological Sequences with Jalview. *Methods Mol. Biol. Clifton NJ* **2231**, 203–224 (2021).

33. PDBe-KB consortium. PDBe-KB: collaboratively defining the biological context of structural data. *Nucleic Acids Res.* **50**, D534–D542 (2022).

34. Xue, H. T., Stanley-Baker, M., Kong, A. W. K., Li, H. L. & Goh, W. W. B. Data considerations for predictive modeling applied to the discovery of bioactive natural products. *Drug Discov. Today* **27**, 2235–2243 (2022).

35. Bludau, I. *et al.* The structural context of posttranslational modifications at a proteome-wide scale. *PLoS Biol.* **20**, e3001636 (2022).

36. Tian, R. *et al.* A Pharmacoinformatics Analysis of Artemisinin Targets and de novo Design of Hits for Treating Ulcerative Colitis. *Front. Pharmacol.* **13**, 843043 (2022).

37. Bordin, N. *et al. AlphaFold2 reveals commonalities and novelties in protein structure space for 21 model organisms*. http://biorxiv.org/lookup/doi/10.1101/2022.06.02.494367 (2022) doi:10.1101/2022.06.02.494367.

38. Cai, S. W. *et al.* Cryo-EM structure of the human CST-Polα/primase complex in a recruitment state. *Nat. Struct. Mol. Biol.* (2022) doi:10.1038/s41594-022-00766-y.

39. Yu, Y. *et al.* Cryo-EM structure of DNA-bound Smc5/6 reveals DNA clamping enabled by multi-subunit conformational changes. *Proc. Natl. Acad. Sci. U. S. A.* **119**, e2202799119 (2022).

40. Armstrong, D. R. *et al.* PDBe: improved findability of macromolecular structure data in the PDB. *Nucleic Acids Res.* gkz990 (2019) doi:10.1093/nar/gkz990.

41. Nair, S. *et al.* PDBe aggregated API: programmatic access to an integrative knowledge graph of molecular structure data. *Bioinformatics* btab424 (2021) doi:10.1093/bioinformatics/btab424.

42. Studer, G. *et al.* QMEANDisCo—distance constraints applied on model quality estimation. *Bioinformatics* **36**, 1765–1771 (2020).

 Click here to access/download;Table;Table 1.docx

Data provider	Model category	Number of structures*
AlphaFill	Template-based	995,411
AlphaFold DB	Ab initio	214,684,311
Genome3D	Template-based	*In progress*
HegeLab	Ab initio	15
isoform.io	Ab initio	48,551
ModelArchive	Ab initio / template-based	1,106
PDBe	Experimentally determined	190,639
PED	Conformation ensembles	275
SASBDB	Experimentally determined	3,912
SWISS-MODEL Repository	Template-based	2,216,915

Table 1 - Members of the 3D-Beacons Network

* Numbers are accurate as of 29th July 2022

Figure 1

Click here to access/download;Figure;Figure 1.png

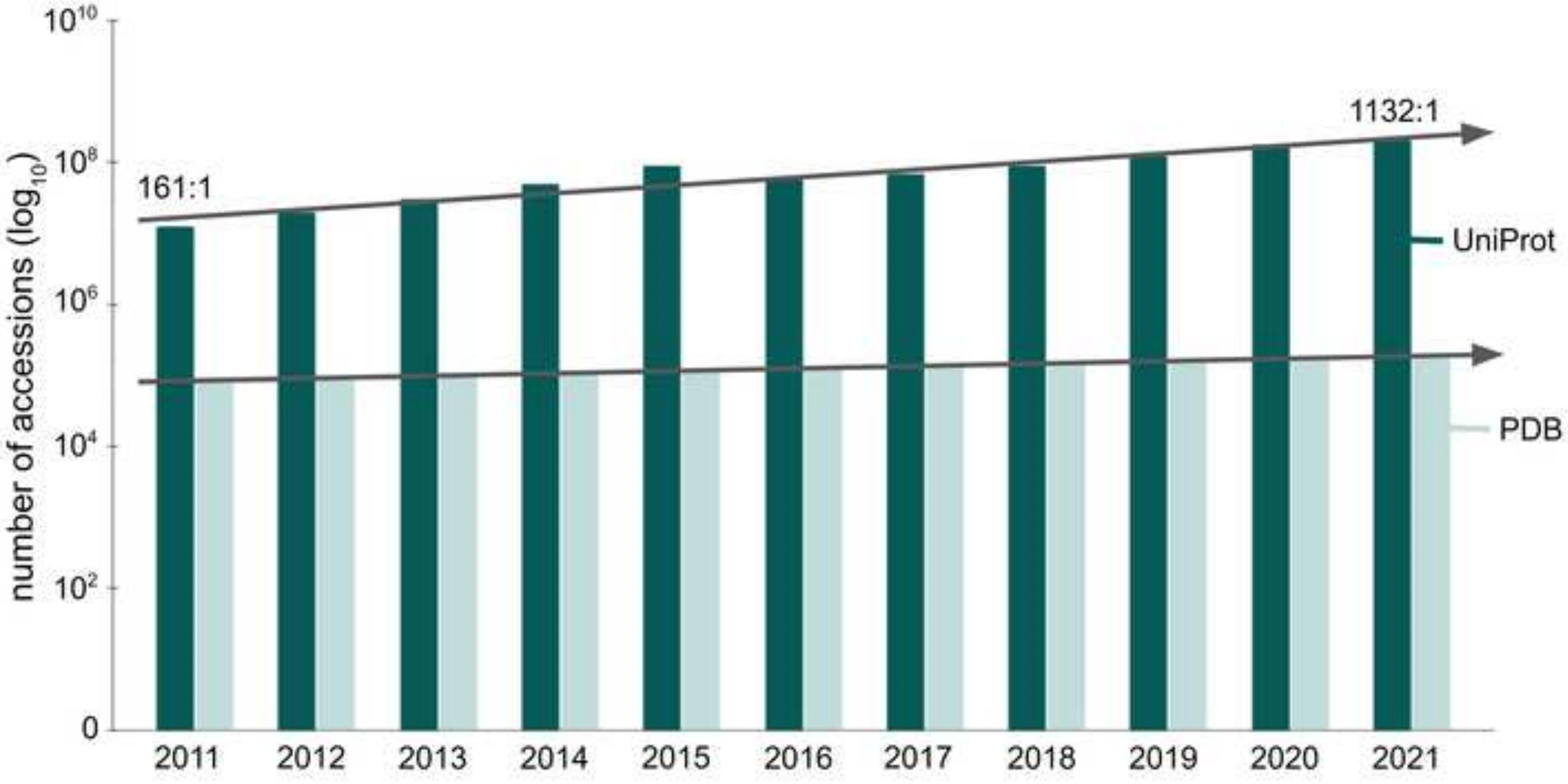

Figure 2

Click here to access/download;Figure;Figure 2.png

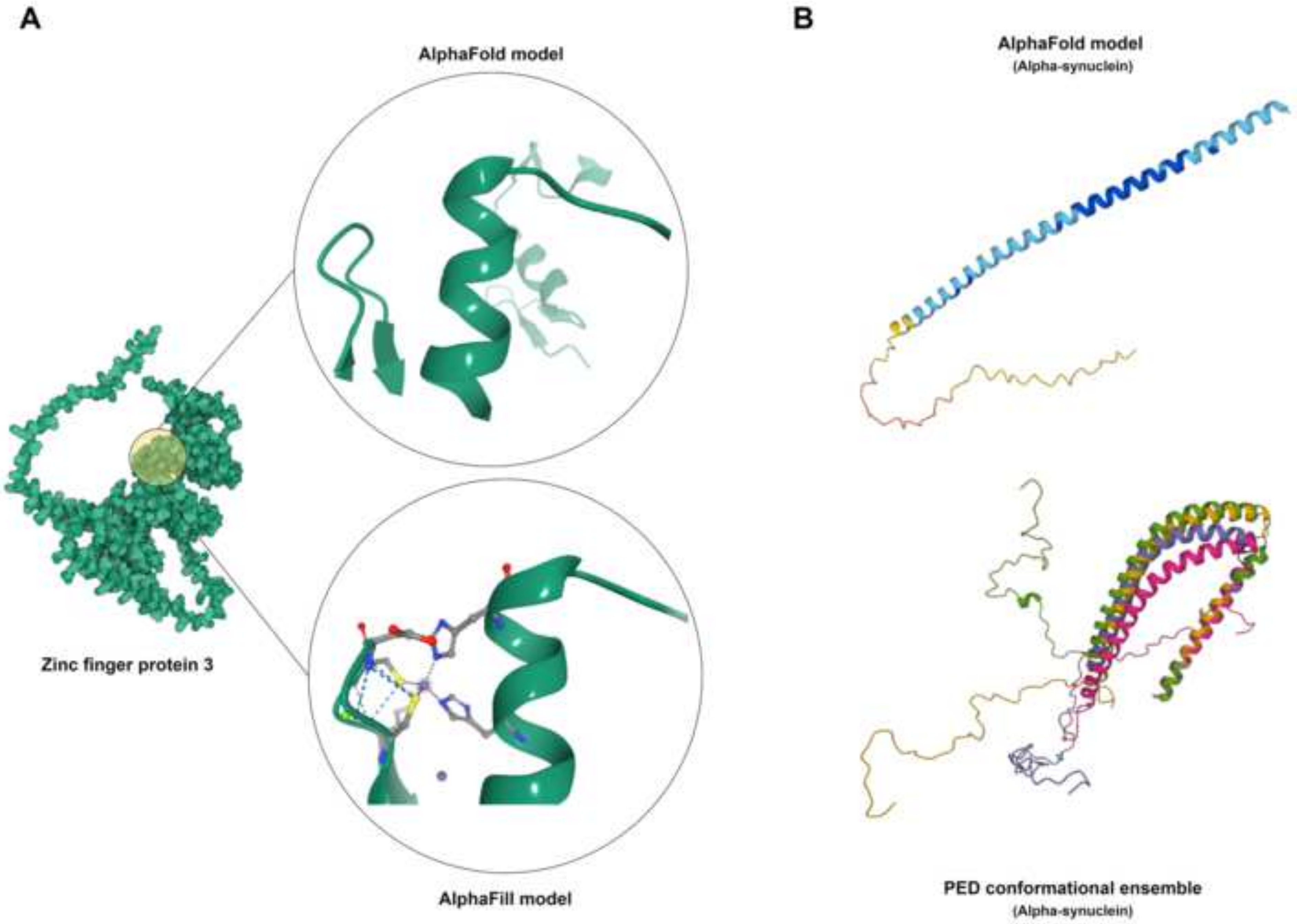

Figure 3

Click here to access/download;Figure;Figure 3.png

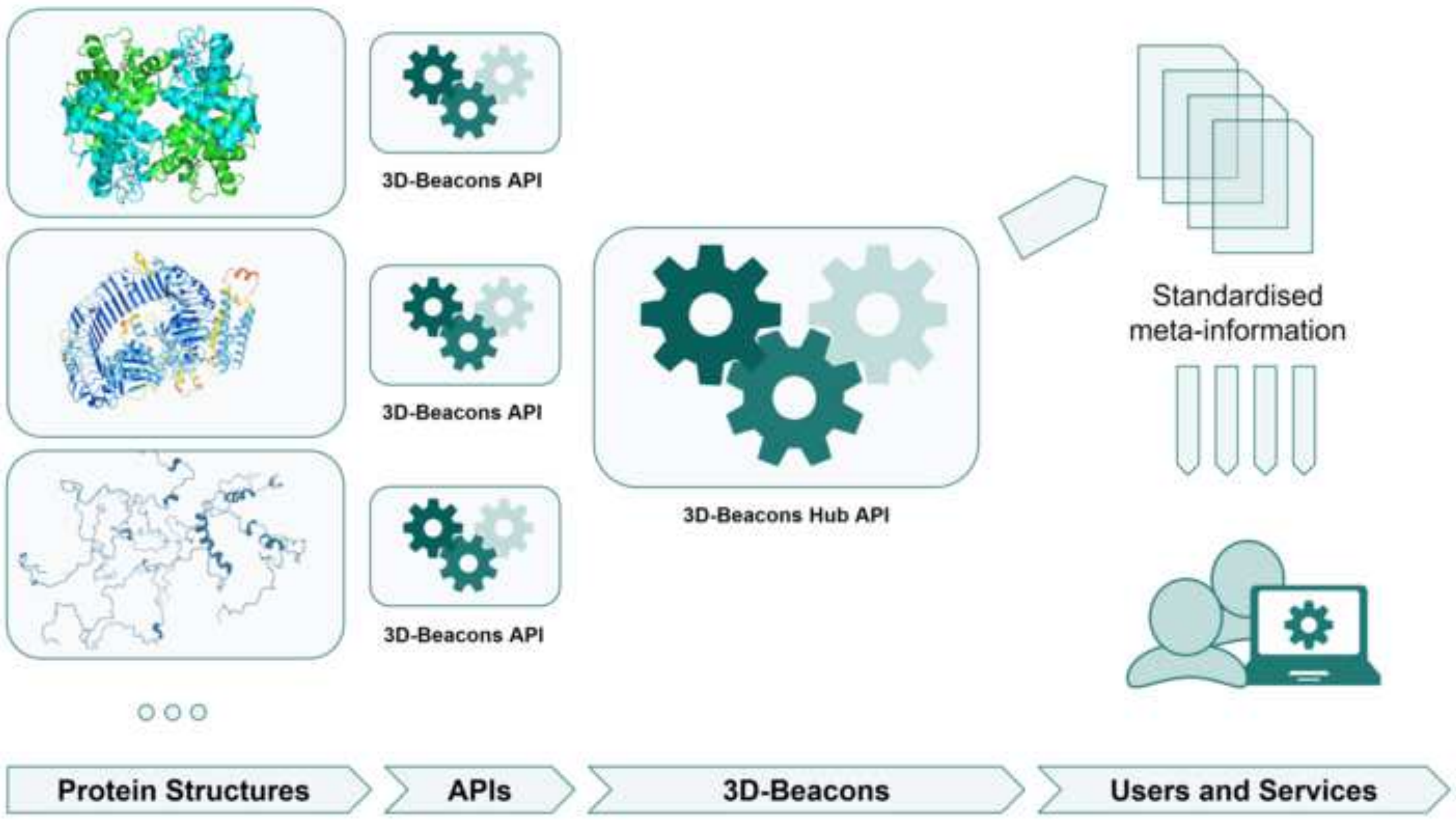

Figure 4 Click here to access/download;Figure;Figure 4.png

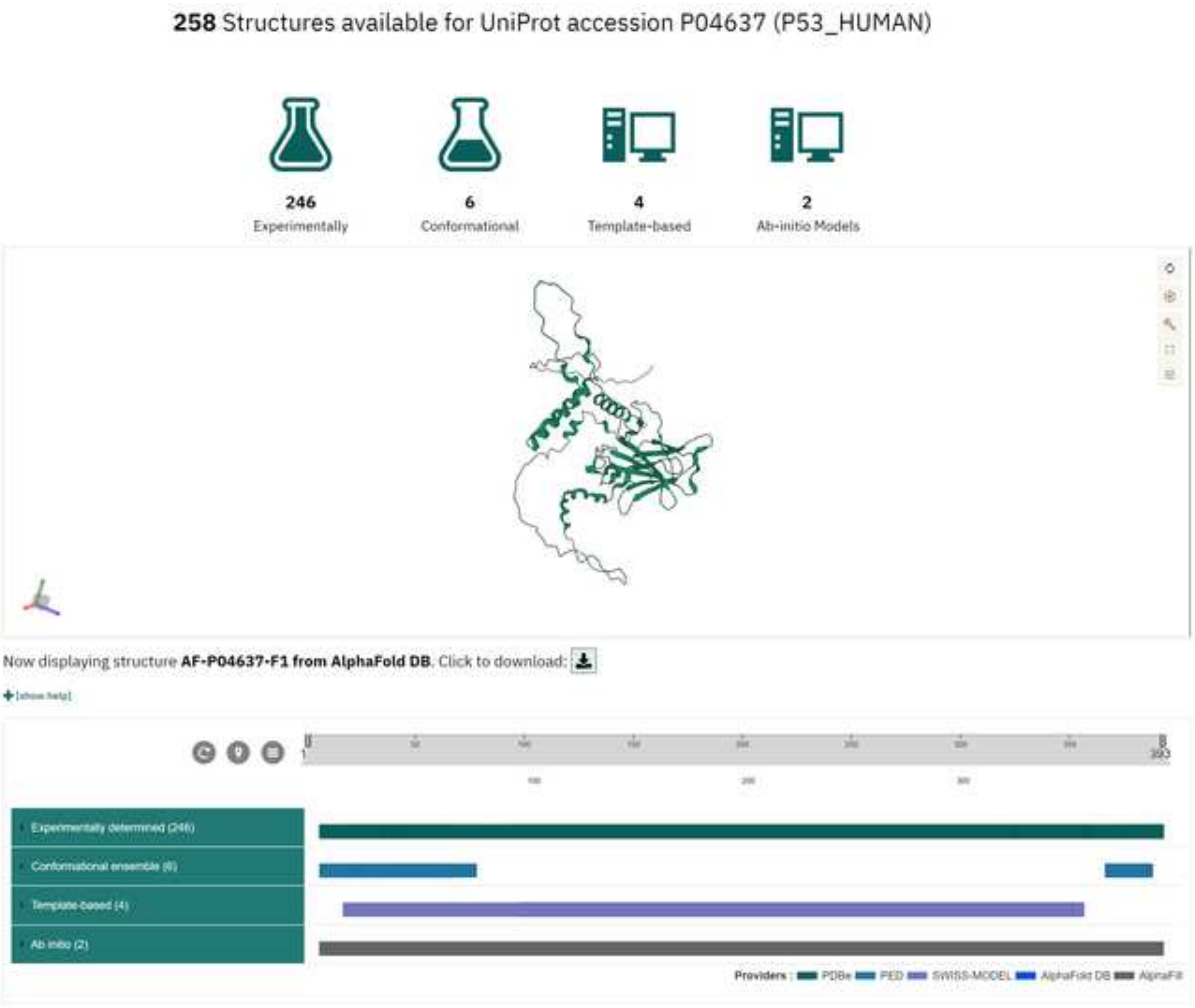

Supplement: giac118_GIGA-D-22-00215_Revision_1 [file giac118_giga-d-22-00215_revision_1.pdf]
