# Supplementary material for: 3D-Beacons: decreasing the gap between protein sequences and structures through a federated network of protein structure data resources
Source: Gigascience. 2022 Nov 30;11:giac118. doi: 10.1093/gigascience/giac118 (PMC9709962; doi:10.1093/gigascience/giac118)
Supplement: giac118_Response_to_Reviewer_Comments_Original_Submission [file giac118_response_to_reviewer_comments_original_submission.pdf]

Author's Response To Reviewer Comments

Close

We thank the reviewers for their positive feedback and their constructive suggestions! Please find our responses below.

Review 1

Q1
1. I recently noticed that RCSB PDB also made it possible to search computational protein models by extending its web interface. The database included ~1 million models from AlphaFold DB and ~1,100 models from ModelArchive, which are main sources of this work as well and are maintained by some of the authors of this work. Even though the number of models and the diversity of the sources accessible via the RCSB PDB interface are fewer than this work, I think the purpose of both works are similar. As there are some overlaps between this work and the RCSB PDB interface in terms of data providers (and authors), what is the significance of this work compared to the RCSB PDB interface?

A1
Thank you for raising this question; we agree it is important to clarify the differences. The recent update in RCSB PDB is based on copying public data and indexing a subset of the data from AlphaFold DB and the ModelArchive. This allowed the developers to embed these models into the RCSB PDB search system. This approach has limitations, such as the lack of transparent data update mechanisms, providing access to incomplete data (i.e. no access to certain confidence metrics) and offering only subsets of data from a subset of data providers. In our approach, we worked together with AlphaFold DB, ModelArchive and other model providers to collaboratively design a federated network that we felt offers a more sustainable and transparent solution to the problem of providing open access to protein structure models. Through 3D-Beacons, all the data providers make all their data accessible, as 3D-Beacons doesn't depend on copying data but rather ensures the data providers can link their own, up-to-date datasets to the network. We added a few sentences to provide similar justification as above.

Q2
2. Most computational models rely on a few data providers, AlphaFold DB, SWISS-MODEL Repository, and AlphaFill (for ligands). In my opinion, it would be better to make the platform richer by recruiting more diverse data providers with different points of view (e.g., conformational ensembles) or different modeling approaches (e.g., machine learning-based approaches with pre-trained protein language models such as OmegaFold). Is there any plan for such progress or promotion of the platform?

A2
Indeed, we envision 3D-Beacons to be an open platform for any data provider can join who agrees to the collaboration guidelines (https://www.ebi.ac.uk/pdbe/pdbe-kb/3dbeacons/guidelines). We have conformational ensembles from the Protein Ensemble Database and SASBDB, but we would welcome more data providers, from small research teams with high quality structures to large datasets from major data resources. Another example of structures we are keen to link, and are in the progress of doing so, is mutant structures from resources such as Missense3D and FoldX. We added a few sentences to make our intentions clearer.

Q3
3. It would be better to have a guide of model selection if there are multiple searched models for an Uniprot ID. Alternatively, providing universal quality assessment scores for models would be an option (by additional data provider). Currently, pLDDT scores are provided, but they are difficult to compare between modeling methods as they were trained independently for each method.

A3
We thank the reviewer for this comment; we are working together with model providers to try and come up with a confidence metric that would allow meaningful ranking of predicted protein structures. We are working on including QMEANDisco in the 3D-Beacons client to provide an assessment tool to data providers who might lack a confidence measure, but this is only a step towards having a unified quality metric we could use to sort/rank the model structures. We added a few sentences to explain this limitation and mention that it is an ongoing effort.

Q4
4. I was able to search on the 3D-Beacons web page a few days ago. However, I could not at the moment of writing these review comments (Sept. 13, 6 p.m. in EDT).

A4
Thank you for reporting this! We will check the traffic logs to see what might have caused the intermittent failure. There is a large-scale compute centre migration happening at EMBL-EBI that affects every EBI-hosted service, which might have caused this temporary outage.

Review 2:

Q5
A minor correction is required on page 7, where the authors describe 4 different types of protein structures: Experimentally determined, Template-based, Ab-initio anc Conformational Ensembles. On many examples available on the website (e.g. https://www.ebi.ac.uk/pdbe/pdbe-kb/3dbeacons/search/P15056), there is one extra category which is structures derived from "Deep learning" methods. I am assuming this comprises a sub-set of Ab-initio structures, which the authors decided to keep as a separate category after submitting this study for publication. The main text should be updated to reflect this change as well as Figure 4.

A5
Thank you very much for raising this inconsistency! This was a bug which we now fixed - There was a major change in the underlying API which included the merging of the categories. This particular update was not yet deployed on the production server - We fixed this, and now the correct categories are visible in the live web pages.

Close
