# Supplementary material for: 3D-Beacons: decreasing the gap between protein sequences and structures through a federated network of protein structure data resources
Source: Gigascience. 2022 Nov 30;11:giac118. doi: 10.1093/gigascience/giac118 (PMC9709962; doi:10.1093/gigascience/giac118)
Supplement: giac118_Response_to_Reviewer_Comments_Revision_3 [file giac118_response_to_reviewer_comments_revision_3.pdf]

Author's Response To Reviewer Comments

Close

Uploaded the missing files

Close
