# Supplementary material for: 3D-Beacons: decreasing the gap between protein sequences and structures through a federated network of protein structure data resources
Source: Gigascience. 2022 Nov 30;11:giac118. doi: 10.1093/gigascience/giac118 (PMC9709962; doi:10.1093/gigascience/giac118)
Supplement: giac118_Reviewer_1_Report_Original_Submission — Lim Heo, Ph.D. -- 9/13/2022 Reviewed [file giac118_reviewer_1_report_original_submission.pdf]

Reviewer Report

Title: 3D-Beacons: Decreasing the gap between protein sequences and structures through a federated network of protein structure data resources

Version: Original Submission **Date:** 9/13/2022

Reviewer name: Lim Heo, Ph.D.

Reviewer Comments to Author:

In this manuscript, authors described a new platform, 3D-Beacons, which is an interface for accessing multiple sources of computational protein models (e.g., AlphaFold DB, SWISS-MODEL) and experimentally determined structures. As the number of protein sequences increases much faster than the growth of experimental structure database (e.g., PDB), computational protein structure models are great alternatives for proteins that do not have experimentally determined structures. Nowadays, many accurate protein models have become available thanks to the progress in template-based modeling techniques for decades and recent advances in de novo protein structure prediction methods using machine-learning approaches. However, those model sources were scattered at their own databases, so there has been difficulties in accessing these models. Thus, in my opinion, the development of a new database or platform, 3D-Beacons, for accessing various computational models is a great movement in the structural biology field. The manuscript well described the description of the platform and some technical details. I have a few minor comments on this work.1. I recently noticed that RCSB PDB also made it possible to search computational protein models by extending its web interface. The database included ~1 million models from AlphaFold DB and ~1,100 models from ModelArchive, which are main sources of this work as well and are maintained by some of the authors of this work. Even though the number of models and the diversity of the sources accessible via the RCSB PDB interface are fewer than this work, I think the purpose of both works are similar. As there are some overlaps between this work and the RCSB PDB interface in terms of data providers (and authors), what is the significance of this work compared to the RCSB PDB interface?2. Most computational models rely on a few data providers, AlphaFold DB, SWISS-MODEL Repository, and AlphaFill (for ligands). In my opinion, it would be better to make the platform richer by recruiting more diverse data providers with different points of view (e.g., conformational ensembles) or different modeling approaches (e.g., machine learning-based approaches with pre-trained protein language models such as OmegaFold). Is there any plan for such progress or promotion of the platform?3. It would be better to have a guide of model selection if there are multiple searched models for an Uniprot ID. Alternatively, providing universal quality assessment scores for models would be an option (by additional data provider). Currently, pLDDT scores are provided, but they are difficult to compare between modeling methods as they were trained independently for each method.4. I was able to search on the 3D-Beacons web page a few days ago. However, I could not at the moment of writing these review comments (Sept. 13, 6 p.m. in EDT).

Level of Interest

Please indicate how interesting you found the manuscript: Choose an item.

Quality of Written English

Please indicate the quality of language in the manuscript: Choose an item.

Declaration of Competing Interests

Please complete a declaration of competing interests, considering the following questions:

- Have you in the past five years received reimbursements, fees, funding, or salary from an organisation that may in any way gain or lose financially from the publication of this manuscript, either now or in the future?
- Do you hold any stocks or shares in an organisation that may in any way gain or lose financially from the publication of this manuscript, either now or in the future?
- Do you hold or are you currently applying for any patents relating to the content of the manuscript?
- Have you received reimbursements, fees, funding, or salary from an organization that holds or has applied for patents relating to the content of the manuscript?
- Do you have any other financial competing interests?
- Do you have any non-financial competing interests in relation to this paper?

If you can answer no to all of the above, write 'I declare that I have no competing interests' below. If your reply is yes to any, please give details below.

I declare that I have no competing interests.

I agree to the open peer review policy of the journal. I understand that my name will be included on my report to the authors and, if the manuscript is accepted for publication, my named report including any attachments I upload will be posted on the website along with the authors' responses. I agree for my report to be made available under an Open Access Creative Commons CC-BY license (http://creativecommons.org/licenses/by/4.0/). I understand that any comments which I do not wish to be included in my named report can be included as confidential comments to the editors, which will not be published.

Choose an item.

To further support our reviewers, we have joined with Publons, where you can gain additional credit to further highlight your hard work (see: https://publons.com/journal/530/gigascience). On publication of this paper, your review will be automatically added to Publons, you can then choose whether or not to claim your Publons credit. I understand this statement.

Yes Choose an item.
