# Supplementary material for: 3D-Beacons: decreasing the gap between protein sequences and structures through a federated network of protein structure data resources
Source: Gigascience. 2022 Nov 30;11:giac118. doi: 10.1093/gigascience/giac118 (PMC9709962; doi:10.1093/gigascience/giac118)
Supplement: giac118_Reviewer_2_Report_Original_Submission — Carlos Rodrigues, PhD -- 9/16/2022 Reviewed [file giac118_reviewer_2_report_original_submission.pdf]

Reviewer Report

Title: 3D-Beacons: Decreasing the gap between protein sequences and structures through a federated network of protein structure data resources

Version: Original Submission **Date:** 9/16/2022

Reviewer name: Carlos Rodrigues, PhD

Reviewer Comments to Author:

This manuscript describes in detail the 3D-Beacons platform/initiative, which aims to facilitate access to 3D data as well as meta-information about experimentally determined and computationally predicted protein structure. This resource is very valuable for the broader scientific community in a time where the number of protein structure data available rapidly increases an many structures may be available for the same protein.A minor correction is required on page 7, where the authors describe 4 different types of protein structures: Experimentally determined, Template-based, Ab-initio anc Conformational Ensembles. On many examples available on the website (e.g. https://www.ebi.ac.uk/pdbe/pdbe-kb/3dbeacons/search/P15056), there is one extra category which is structures derived from "Deep learning" methods. I am assuming this comprises a sub-set of Ab-initio structures, which the authors decided to keep as a separate category after submitting this study for publication. The main text should be updated to reflect this change as well as Figure 4.

Level of Interest

Please indicate how interesting you found the manuscript: Choose an item.

Quality of Written English

Please indicate the quality of language in the manuscript: Choose an item.

Declaration of Competing Interests

Please complete a declaration of competing interests, considering the following questions:

- Have you in the past five years received reimbursements, fees, funding, or salary from an organisation that may in any way gain or lose financially from the publication of this manuscript, either now or in the future?
- Do you hold any stocks or shares in an organisation that may in any way gain or lose financially from the publication of this manuscript, either now or in the future?
- Do you hold or are you currently applying for any patents relating to the content of the manuscript?
- Have you received reimbursements, fees, funding, or salary from an organization that holds or has applied for patents relating to the content of the manuscript?

- Do you have any other financial competing interests?
- Do you have any non-financial competing interests in relation to this paper?

If you can answer no to all of the above, write 'I declare that I have no competing interests' below. If your reply is yes to any, please give details below.

I declare that I have no competing interests

I agree to the open peer review policy of the journal. I understand that my name will be included on my report to the authors and, if the manuscript is accepted for publication, my named report including any attachments I upload will be posted on the website along with the authors' responses. I agree for my report to be made available under an Open Access Creative Commons CC-BY license (http://creativecommons.org/licenses/by/4.0/). I understand that any comments which I do not wish to be included in my named report can be included as confidential comments to the editors, which will not be published.

Choose an item.

To further support our reviewers, we have joined with Publons, where you can gain additional credit to further highlight your hard work (see: https://publons.com/journal/530/gigascience). On publication of this paper, your review will be automatically added to Publons, you can then choose whether or not to claim your Publons credit. I understand this statement.

Yes Choose an item.
